# Loss of the E3 ubiquitin ligase MARCHF6 alters hepatic lipid metabolism and drives spontaneous hepatosteatosis

**DOI:** 10.1016/j.molmet.2026.102379

**Published:** 2026-05-02

**Authors:** Vinay Sachdev, Nienke M. van Loon, Jenina Kingma, Roelof Ottenhoff, Josephine M.E. Tan, Marlene van den Berg, Suzanne Duijst, Aldo Jongejan, Johannes H.M. Levels, Patrick C.N. Rensen, Sander Kooijman, Jan-Freark de Boer, Folkert Kuipers, Katharina B. Kuentzel, Dagmar Kratky, Yun Kwon, Anja Zeigerer, Sebastian Hendrix, Noam Zelcer

**Affiliations:** 1 Department of Medical Biochemistry, Amsterdam UMC location AMC, University of Amsterdam, Amsterdam, the Netherlands; 2 Amsterdam Gastroenterology, Endocrinology, and Metabolism Institute, Amsterdam UMC, the Netherlands; 3 Amsterdam Cardiovascular Sciences Institute, Amsterdam UMC, the Netherlands; 4 Department of Physiology, Amsterdam UMC location VUMC, Free University Amsterdam, Amsterdam, the Netherlands; 5 Tytgat Institute for Liver and Intestinal Research, Amsterdam UMC location AMC, University of Amsterdam, Amsterdam, the Netherlands; 6 Bioinformatics Laboratory, Department of Epidemiology & Data Science, Amsterdam UMC location AMC, Amsterdam, the Netherlands; 7 Department of Experimental Vascular Medicine, Amsterdam UMC location AMC, University of Amsterdam, Amsterdam, the Netherlands; 8 Department of Medicine, Division of Endocrinology & Einthoven Laboratory for Experimental Vascular and Regenerative Medicine, Leiden University Medical Center, Leiden, the Netherlands; 9 Department of Pediatrics, University of Groningen, University Medical Center Groningen, Groningen, the Netherlands; 10 Department of Laboratory Medicine, University of Groningen, University Medical Center Groningen, Groningen, the Netherlands; 11 European Research Institute for the Biology of Ageing (ERIBA), University of Groningen, University Medical Center Groningen, Groningen, the Netherlands; 12 Gottfried Schatz Research Center, Molecular Biology and Biochemistry, Medical University of Graz, Graz, Austria; 13 BioTechMed-Graz, Graz, Austria; 14 European Center for Angioscience (ECAS), Medical Faculty Mannheim, Heidelberg University, Mannheim, Germany; 15 Institute for Diabetes and Cancer, Helmholtz Center Munich, Neuherberg, Germany; 16 Joint Heidelberg-IDC Translational Diabetes Program, Inner Medicine 1, Heidelberg University Hospital, Heidelberg, Germany; 17 German Center for Diabetes Research (DZD), Neuherberg, Germany

**Keywords:** MASLD, SREBP, MARCHF6, Lipogenesis, Post-transcriptional regulation, NADPH

## Abstract

Metabolic dysfunction-associated steatotic liver disease (MASLD) and its progressive form, steatohepatitis (MASH), feature excessive hepatic fat accumulation, yet the relative contributions of dietary vs. endogenous fats and their interactions has remained enigmatic. Here, we identify the endoplasmic reticulum–associated E3 ubiquitin ligase MARCHF6 as a pivotal regulator of hepatic lipid metabolism. Global or hepatocyte-specific deletion of *Marchf6* induced spontaneous accumulation of triglycerides and cholesteryl esters under chow-fed conditions, revealing a cell-autonomous hepatic defect independent of caloric excess. Loss of MARCHF6 stabilized its substrate squalene epoxidase (SQLE), enhancing sterol pathway flux while concomitantly activating the SREBP1-associated lipogenic transcriptional program and increasing lipoprotein clearance. Accordingly, lipidomic analyses demonstrated remodeling of the hepatic lipidome towards polyunsaturated, long-chain neutral lipids, consistent with increased lipogenesis-driven NADPH consumption. In line with this, pharmacological inhibition of the oxidative pentose phosphate pathway reduced lipid accumulation in MARCHF6-deficient human hepatocytes. Congruently, transcriptomic data from human MASLD/MASH patients revealed reduced hepatic *MARCHF6* expression alongside an increase in that of the lipogenic genes *SREBF1*, *FASN*, and *SCD1*. Overall, these data establish MARCHF6 as a multifaceted gatekeeper that integrates sterol turnover, NADPH usage, and lipogenesis to maintain hepatic lipid homeostasis.

## Highlights

• MARCHF6 loss leads to lipid accumulation in mouse livers and human hepatocytes.
• Hepatic lipid accumulation is mainly fueled by increased lipid uptake and synthesis.
• Ablation of MARCHF6 increases triglyceride chain length and desaturation.
• MARCHF6 deletion augments hepatic NADPH consumption.
• Expression of MARCHF6 is decreased in MASLD patient samples.

## Graphical abstract

**Figure:**
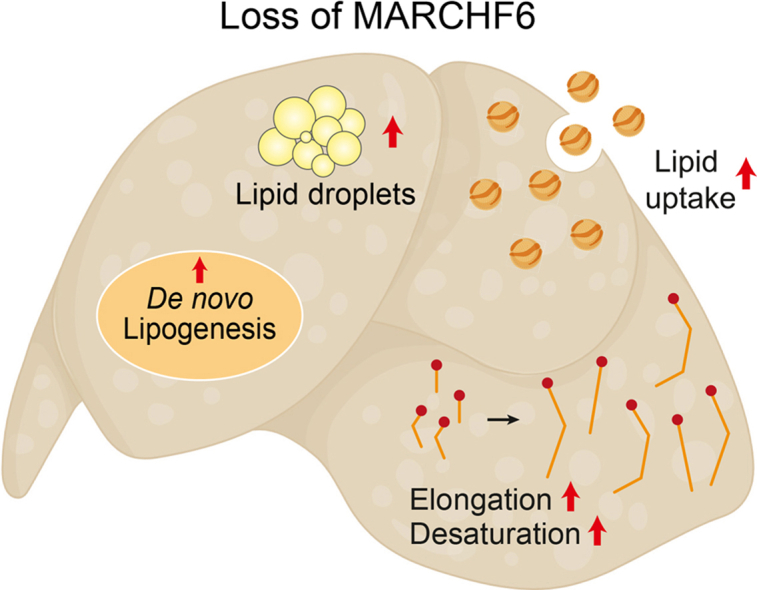

## 1 Introduction

Metabolic dysfunction-associated steatotic liver disease (MASLD), characterized by the accumulation of triglycerides (TG) and cholesteryl esters in the liver, is considered to represent the hepatic manifestation of the metabolic syndrome [1,2]. Common risk factors that are associated with developing the metabolic syndrome like a high caloric diet, a sedentary lifestyle, and insulin resistance also underlie the development of MASLD. In recent years, studies have implicated genetic variation in, amongst others, *TM6SF2* [3]*, PNPLA3* [4], *GCKR* [5], *MBOAT7* [6]*,* and *HSD17β13* [7] in MASLD development. MASLD is associated with an increased cardiovascular risk and, importantly, can gradually progress to metabolic dysfunction-associated steatohepatitis (MASH), liver cirrhosis, and ultimately hepatocellular carcinoma in some individuals [8]. The molecular events driving progression of MASLD towards cirrhosis and hepatocellular carcinoma are not fully understood. Yet a growing body of research points towards dysregulation of hepatic lipid metabolism as a contributing factor in this detrimental transformation.

Hepatic lipid metabolism is governed by the sterol-regulatory element binding proteins (SREBPs) [9], of which three isoforms exist, namely SREBP1a and SREBP1c, which are transcribed from the same gene yet use a different transcriptional start site [10], and SREBP2 [11]. In the liver, SREBP1c is the prominent SREBP1 isoform and primarily controls fatty acid synthesis and mobilization, while SREBP2 controls cholesterol biosynthesis and lipoprotein uptake [12,13]. Both are produced as precursor membrane proteins in the endoplasmic reticulum (ER) and in response to metabolic cues undergo regulated trafficking to the Golgi where they are proteolytically activated to release their transcriptionally active domain [9,14]. SREBP activation is subject to exquisite regulation and given their central role, a multitude of pathways and processes converge on them to regulate their activation. In recent years it has become apparent that ER-resident E3 ubiquitin ligases contribute to the regulation of SREBP activation [15]. This regulation can be direct, by promoting the ubiquitylation and subsequent degradation of SREBPs, as has been proposed for the E3 ligases TRC8 [16], HRD1 [17], RNF20 [18] and FBX7 [19]. Additionally, several ER-resident E3 ligases have been demonstrated to modulate the SREBP pathway. Examples for that are GP78, TRC8, RNF145 and HDR1 regulating 3-hydroxy-3-methylglutaryl coenzyme A reductase (HMGCR) [20], the rate-limiting enzyme in cholesterol synthesis, or RNF145 controlling the trafficking of SCAP (SREBP cleavage-activating protein) [21].

We and others have recently identified the ERAD-associated E3 ubiquitin ligase MARCHF6 as a post-transcriptional regulator of squalene epoxidase (SQLE), the enzyme in cholesterol biosynthesis that commits the mevalonate pathway to sterol production [22,23]. Working together with its cognate E2 conjugating enzyme UBE2J2 [24], MARCHF6 controls cholesterol biosynthesis by coupling the level of the end-product, cholesterol, to ubiquitylation and subsequent degradation of SQLE. In recent years, the degradation targets of MARCHF6 have been expanded to other enzymes in the mevalonate pathway, including DHCR24, LDM, SC4MOL, and SC5D [[25], [26], [27]]. Notably, a common feature of these enzymes is the consumption of NADPH as part of their enzymatic action [28]. Therefore, it is intriguing that a recent study implicated MARCHF6 as a sensor for cellular NADPH levels owing to residues 870–900 in its C-terminal MARCHF6-regulatory region (MARA) domain [29]. Sensing of NADPH controls the degradation rate of SQLE by MARCHF6, promoting the idea that MARCHF6 couples sensing of the cellular redox state to the control of cholesterol biosynthesis [28].

The liver is the central organ controlling systemic lipid metabolism, and many of the enzymatic steps involved in the synthesis of both fatty acids and cholesterol require NADPH as a cofactor. Previously, we reported that in hepatocyte-like HepG2 cells the activity of MARCHF6 controls the balance between cholesterol synthesis and uptake via the low density-lipoprotein receptor (LDLR) pathway [30]. Furthermore, in HeLa, HEK293T, and HepG2 cells MARCHF6 was also reported to regulate the level of PLIN2, a lipid droplet (LD)-associated protein implicated in hepatic lipid metabolism [31,32]. These studies were limited to cultured cells, and whether MARCHF6 is a physiological regulator of hepatic and systemic lipid metabolism is unknown at present. To address this gap, we generated and characterized mice with global- and hepatocyte-specific ablation of *Marchf6.* We show that loss of hepatic *Marchf6* has dramatic consequences on hepatic lipid metabolism owing to dysregulated clearance of plasma lipoproteins and *de novo* lipogenesis (DNL).

## 2 Materials and methods

### 2.1 Cell line culture

HepG2 and HEK293 cells (ATCC) were cultured at 37 °C and 5% CO_2_ in a humidified incubator. Cells were grown in Dulbecco's Modified Eagle Medium (DMEM, Gibco) supplemented with 4.5 g/L glucose, 0.86 g/L GlutaMAX, 10% fetal bovine serum (FBS), penicillin (100 units/mL) and streptomycin (100 μg/mL). All other reagents used in this study are listed in Supplemental Table 1.

### 2.2 Isolation and culture of primary hepatocytes

#### 2.2.1 Primary mouse hepatocytes

Cells were isolated from mice using a two-step perfusion method as described by Gilglioni et al. [33]. Briefly, livers were cannulated via the portal vein and perfused at 37 °C with calcium-free Krebs buffer containing 23.9 mM NaHCO_3_ and 10 mM HEPES at a flow rate of 3.5 mL/min for 10 min. The buffer was then supplemented with collagenase type IV (100 U/ml, Merck) and 2.4 mM CaCl_2_ and perfusion was continued for an additional 8 min. All perfusion buffers were pre-equilibrated with 95% O_2_/5% CO_2_ to ensure proper oxygenation. Digested livers were transferred to a sterile cell culture dish, submerged in calcium-containing Krebs buffer, mechanically dispersed, and filtered through a 70 μm cell strainer to remove cell aggregates. The cell suspension was centrifuged at 50×*g* for 2 min at 4 °C and washed three times with calcium-containing Krebs buffer supplemented with 0.2 % bovine serum albumin (BSA). Subsequently, cells were resuspended in DMEM containing 10% FBS, 1 g/L glucose, 26 mM sodium bicarbonate, 100 nM dexamethasone (Sigma; DEX), 100 nM insulin (Gibco), penicillin (100 U/mL) and streptomycin (100 μg/mL). Cell number and viability were determined by trypan blue exclusion and cells were seeded onto collagen-coated six-well plates. After cell attachment, the medium was refreshed with the same medium without FBS, and experiments were performed within 48 h of seeding.

#### 2.2.2 Primary human hepatocytes

Cryopreserved primary human hepatocytes (PHH) from a Hispanic male donor were used for all experiments, as previously described [34]. Thawed hepatocytes were rapidly warmed at 37 °C for 45 s and immediately resuspended in pre-warmed William's E medium (WE) without FBS. After centrifugation at 50×*g* for 10 min, the cell pellet was resuspended in 5 mL pre-warmed WE medium containing 100 μM DEX. Cell viability and number were determined prior to seeding. A total of 2 × 10^5^ cells were plated per well in collagen-coated 24-well plates using WE supplemented with 10% FBS, 5% penicillin-streptomycin, and 100 μM DEX. Cells were incubated at 37 °C with 5% CO_2_ for 3 h to allow for cell adhesion, followed by two washes with PBS. A second collagen layer was then applied to establish the collagen sandwich (3D) culture system. The maintenance medium (containing 100 nM DEX) was replenished daily.

### 2.3 CRISPR/Cas9-mediated ablation of MARCHF6 in HepG2 cells

The first exon of *MARCHF6* was targeted using two independent sgRNAs (guide 1: fw 5′-CACCGCGCCGACTTACCTTCCTCCG-3′, rv 5′-AAACCGGAGGAAGGTAAGTCG GCGC-3′; guide 2: fw 5′-CACCGAAGATGGACACCGCGGAGGA-3′, rv 5′-AAACTCCTC CGCGGTGTCCATCTTC-3′) or a control sgRNA that targets the *AAVS1* safe-harbor locus. Annealed oligos were cloned into the lentiCRISPRv2 plasmid (Addgene # 52961). Lentiviral particles were produced in HEK293T cells as previously described [35]. Briefly, HEK293T cells were transfected with lentiCRISPRv2 along with third-generation lentiviral packaging constructs to generate viral particles. HepG2 cells were subsequently transduced for 16 h with lentiviral particle-containing supernatant supplemented with complete DMEM (4:1 ratio) and 10 μg/mL polybrene. Single cell colonies were selected using 2 μg/mL puromycin (Sigma) and expanded for use in this study.

### 2.4 Generation of constitutive and hepatocyte-specific Marchf6^KO^ mice

Conditional *Marchf6* mice on a C57Bl/6J background (*Marchf6*^*[fl/fl]*^) were generated by genOway (Lyon, France). A schematic representation of the knock-out strategy is provided in Supplementary Fig. 1. In brief, a targeting vector containing a long homology region of 6063 kb and a short homology region of 1653 kb, two loxP sites flanking the third exon of *Marchf6* (which encodes the RING domain of MARCHF6), a neomycin gene flanked by FRT sites for positive selection and a diphteria toxin A negative selection marker was used. The linearized targeting vector was transfected into C57BL/6 ES cells (genOway, Lyon, France) through electroporation (260Volt, 500 μF) of 108 ES cells in the presence of 100 μg of linearized plasmid. Positive selection was initiated post 48 h after electroporation by addition of 200 μg/mL of G418 (150 μg/mL of active component, Life Technologies, Inc.). Resistant single homologous clones were selected following PCR and Southern blot analysis. Clones were microinjected into recipient blastocysts derived from albino C57BL/6J-Tyrc-2J/J mice, to generate chimeras of over 50%. Chimeric male mice were bred with either Cre deleter mice to generate heterozygous *Marchf6* mice, in which exon 2 of one allele of *Marchf6* was excised constitutively *(Marchf6*^*(+/−)*^*)* or with Flp deleter mice to generate conditional *Marchf6* mice, in which the second exon of *Marchf6* was floxed. The latter were interbred to create homozygous conditional *Marchf6* mice (*Marchf6*^*(fl/fl)*^). Hepatocyte-specific *Marchf6* knockout mice were generated by crossing *Marchf6*^*(fl/fl)*^ mice with mice expressing Cre under the albumin promotor (AlbuminCre^+^ mice, kind gift of Dr R. Oude Elferink, Amsterdam UMC, The Netherlands). The resulting *Marchf6*^*(fl/fl)*^*AlbuminCre*^*+*^ (M6^LKO^) and *Marchf6*^*(fl/fl)*^*AlbuminCre*^*-*^ (WT) mice were interbred to generate mice for the reported experiments.

### 2.5 Animal experiments

All experiments were approved by the local animal welfare committee of the AMC and performed in accordance with national regulations and guidelines (DBC292 and AVD11800202317543). Mice were group housed under SPF conditions with a 12 h light/dark cycle and temperature-controlled environment in individually ventilated cages with ad libitum access to drinking water and food. Unless otherwise stated, male littermates were randomly assigned to the experimental groups and fasted for 4 h before sacrifice. Mice were euthanized by *intraperitoneal* (*i.p.)* administration of 238 mg ketamine/kg mouse and 23.8 mg xylazine/kg mouse, blood was collected, and plasma isolated by centrifugation at 1,500×*g* for 7 min at 4 °C. Livers and other organs were rapidly excised, weighed, and immediately frozen in liquid nitrogen unless stated otherwise and stored at −80 °C. ***Indirect calorimetry and lipoprotein uptake***: Male *Marchf6*^LKO^ and littermate controls (N = 8/group) were fed a chow (Teklad 2916) diet. At the age of 11 weeks, mice were individually housed in metabolic cages (Promethion line, Sable Systems, Promethion line, Las Vegas, NV, USA) for 1 week to assess O_2_ consumption, CO_2_ production, and food/water intake. Subsequently, mice were fed a high fat diet (HFD, Ssniff D14292) for an additional 3 weeks. Subsequently, mice were fasted for 4 h and then received a bolus of VLDL-like particles labelled with glycerol tri[^3^H]oleate (Revvity) and [^14^C]cholesteryl oleate (Revvity) by tail vein injection, as we previously described [36]. Blood was sampled from the tail vein at the indicated time points after injection. At the end of the experiment the mice were euthanized, perfused with ice-cold PBS and organs were harvested. Tissue samples were dissolved overnight at 55 °C in 0.5 mL Solvable (Revvity). All samples were mixed with Ultima Gold scintillation fluid (Revvity) before radioactivity was quantified using a Tri-Carb 2910 TR Low Activity Liquid Scintillation Analyzer (PerkinElmer). ***VLDL secretion assay***: To evaluate hepatic VLDL secretion 10-week-old male *Marchf6*^LKO^ and control littermate mice (N = 5/group) were fasted for 4 h, injected intraperitoneally with Poloxamer 470 (1 g/kg) and blood samples were collected at the indicated time points. Samples were kept on ice, spun down at 1,500×*g* for 7 min at 4 °C to isolate plasma. Plasma TG content (Diagnostic Systems) and total cholesterol (Biolabo) content were determined using commercial kits according to the manufacturers’ instructions. ***Glucose tolerance test:*** Glucose tolerance test (GTT) was performed on 19-weeks-old female mice. Mice were fasted for 4 h and then 2 g/kg glucose was administered orally. Tail vein blood was collected at the indicated time points and blood glucose levels were measured.

### 2.6 Plasma lipoprotein analysis by FPLC

Total cholesterol and TG content in the VLDL, LDL and HDL lipoprotein classes were determined as previously described [37]. Briefly, a system consisting of a PU-4180 quaternary pump with a DG-4000 inline degasser and a Diode Array Detector (DAD MD 4015, Jasco) was used. Lipoproteins were separated on a Superose 6 increase 10/30 column (GE Healthcare) and subsequently eluted using Tris-buffered saline (pH 7.4). An auxiliary (PU-4080i Plus, Jasco) was used for in-line injection of cholesterol PAP or TG enzymatic substrate reagent (Sopachem) to quantify the total cholesterol and TG content of the separated lipoprotein fractions.

### 2.7 Hepatic lipid extraction and measurement

Snap frozen tissues were mechanically disrupted in 1 mL RIPA buffer supplemented with phenylmethylsulfonyl fluoride (PMSF, Sigma) and protease inhibitors (PI) using a Magnalyser (Roche). Subsequently, lipids were extracted following Folch's method. Briefly, 100 μL of lysate were transferred to a glass tube, and 3 mL of a 2:1 mixture chloroform:methanol was added and incubated overnight under constant shaking. Next, 750 μL water were added and mixed thoroughly buy vortexing and then spun down for 5 min at 1000×*g*. The lower, organic phase containing the lipids was then transferred to a fresh glass tube. Samples were dried under nitrogen gas and the remaining pellet dissolved in 100 μL of water with 2% (v/v) Triton X-100 at 37 °C. Cells were grown in a 10-cm^2^ dish, harvested in 1 mL RIPA supplemented with PMSF and PI, and lipids were extracted as described above. TG and cholesterol concentrations were determined using kits following the manufacturers' instructions (Diasys and Biolabo). The concentration was normalized to the protein concentration in the lysate using the BCA assay (Thermo Fisher) according to manufacturer's instructions.

### 2.8 Lipid droplet analysis in primary human hepatocytes

Primary human hepatocytes (PHH) were plated and cultured on cover slips (14 mm Ø, Thickness 1 round 0.13). siRNA transfection was performed using RNAiMAX reagent according to manufacturer's instructions. Cells were transfected with 40 nM siMARCHF6 or control siRNA (siCTR) for 6 h. On day 5 after transfection, cells were treated with 10 μM of either NB-598 (squalene epoxidase inhibitor) or 6-aminonicotinamide (6-AN, pentose phosphate pathway inhibitor) for 24 h. As a positive control, PHHs were loaded with free fatty acids by culturing them in WE medium containing 10% FBS, 5% penicillin-streptomycin, and a combination of BSA-conjugated free fatty acids (0.15 mM; BSA:FFA molar ratio of 1:5). On day 6, cells were fixed with 4% paraformaldehyde (PFA) at room temperature for 30 min, washed twice in PBS and permeabilized with 0.1% Triton X-100 for 1 h. Following permeabilization, cells were blocked for 2 h in 10% horse serum (Thermo Fisher Scientific). Then PHHs were washed twice for 4 h in TNT (10 mM) Tris–HCl pH 8.0, 300 mM NaCl and Tween-20 (0.1% v/v) and subsequently incubated with BODIPY 493/503 (1:1000; Thermo Fisher) in 5% horse serum overnight at 4 °C. Finally, PHHs were washed twice in PBS, incubated with DAPI (1:10,000; Thermo Fisher) for 5 min and mounted onto glass slides using 0.1 g/mL MOWIOL 4–88 (Merck). Immunofluorescent samples were analyzed using a laser scanning confocal microscope (Olympus FluoView 1200; Olympus Corporation) equipped with an Olympus UPlanSAPO × 60 1.35 solid immersion lens oil immersion objective (Olympus) at a resolution of approximately 100 μm pixel−1 ( × 60) and 600 nm step size. Image analysis was performed for individual images after background subtraction with a minimum of 10 cells per technical replicate using Fiji software (ImageJ, v.2.0.0-rc-69/1.51w). For background subtraction, the Fiji rolling ball function (radius 50) was applied. To quantify LDs in PHH, a built in Fiji plug-in for particle analysis was used. Fluorescent LDs with a pixel^2^ from 0.1 to infinity and circularity from 0.00 to 1.00 were included. Mean size, occupancy, and number of LDs was evaluated in individual cells.

### 2.9 Omics analysis of liver tissue

#### 2.9.1 Transcriptomics

Sequencing and bioinformatic analysis were performed by the core facility Genomics and Bioinformatics Core Unit of the Amsterdam UMC, Amsterdam, The Netherlands as previously described [38]. Total RNA was isolated using the Direct-zol RNA miniprep kit (Zymo Research), quantified using a Qubit 3 Fluorimeter (Invitrogen), reverse transcribed into cDNA to generate an Illumina indexed mRNA library using a KAPA mRNA hyperPrep kit (KAPA Biosystems). Sequencing was performed on an Illumina HiSeq4000 platform. Reads were subjected to quality control (FastQC v0.11.15, Picard Tools), trimmed using Trimmomatic v0.36 [39] and aligned to the genome (Ensembl v93) using HISAT2 (v2.1.0) [40]. Counts were obtained using HTSeq (v0.6.1) [41] using the corresponding GTFs. Statistical analyses were performed using the edgeR [42] and limma/voom [43] R packages*.* All genes with no counts in any of the samples were removed whilst genes with more than 2 reads in at least 6 of the samples were kept. Count data was transformed to log2-counts per million (logCPM), normalized by applying the trimmed mean of M-values method [42] and precision weighted using voom [44]. Differential expression was assessed using an empirical Bayes moderated t-test within limma's linear model framework including the precision weights estimated by voom. Resulting *p* values were corrected for multiple testing using the Benjamini-Hochberg false discovery rate. Genes were re-annotated with biomaRt using the Ensembl genome databases (*v93*). Geneset enrichment was performed with MSigDB genesets (v6.1) using CAMERA approach as implemented in limma. Genesets were mapped to mouse genes using Homologene build 68 (downloaded from ftp://ftp.ncbi.nih.gov/pub/HomoloGene/). Analysis was performed using R v3.5.0 and Bioconductor v3.7.

#### 2.9.2 Lipidomics

Lipidomic analysis of samples was performed at the Core Facility Metabolomics of the Amsterdam UMC, Amsterdam, the Netherlands (www.cfmetabolomics.nl), essentially as recently described [45]. Briefly, 25 μL of plasma samples were mixed with the following amounts of internal standards dissolved 1:1 (v/v) in methanol:chloroform: Bis(monoacylglycero)phosphate BMP(14:0)2 (0.2 nmol), Ceramide-1-phosphate C1P (d18:1/12:0) (0.127 nmol), D_7_-Cholesteryl Ester CE(16:0) (2 nmol), Ceramide Cer(d18:1/12:0) (0.118 nmol), Ceramide Cer(d18:1/25:0) (0.130 nmol), Cardiolipin CL(14:0)4 (0.1 nmol), Diacylglycerol DAG(14:0)2 (0.5 nmol), Glucose Ceramide GlcCer(d18:1/12:0) (0.126 nmol), Lactose Ceramide LacCer(d18:1/12:0) (0.129 nmol), Lysophosphatidicacid LPA(14:0) (0.1 nmol), Lysophosphatidylcholine LPC(14:0) (0.5 nmol), Lysophosphatidylethanolamine LPE(14:0) (0.1 nmol), Lysophosphatidylglycerol LPG(14:0) (0.02 nmol), Phosphatidic acid PA(14:0)2 (0.5 nmol), Phosphatidylcholine PC(14:0)2 (2 nmol), Phosphatidylethanolamine PE(14:0)2 (0.5 nmol), Phosphatidylglycerol PG(14:0)2 (0.1 nmol), Phosphatidylinositol PI(8:0)2 (0.5 nmol), Phosphatidylserine PS(14:0)2 (5 nmol), Sphinganine 1-phosphate S1P(d17:0) (0.124 nmol), Sphinganine-1-phosphate S1P(d17:1) (0.125 nmol), Ceramide phosphocholines SM(d18:1/12:0) (2.129 nmol), Sphingosine SPH(d17:0) (0.125 nmol), Sphingosine SPH(d17:1) (0.125 nmol), Triacylglycerol TAG(14:0)2 (0.5 nmol). Next, per sample, 1.5 mL 1:1 (v/v) methanol:chloroform was added per sample and after thoroughly mixing, the samples were centrifuged for 10 min at 20,000×*g*. The supernatant was transferred to a glass vial and evaporated under a stream of nitrogen at 60 °C. The residue was dissolved in 150 μL of 1:1 (v/v) methanol:chloroform. Lipids were analyzed using a Thermo Scientific Ultimate 3000 binary HPLC coupled to a Q Exactive Plus Orbitrap mass spectrometer. For normal phase separation, 2 μL of each sample was injected onto a Phenomenex® LUNA silica, 250 ∗ 2 mm, 5 μm 100 Å. Column temperature was held at 25 °C. Mobile phase consisted of (A) 85:15 (v/v) methanol:water containing 0.0125% formic acid and 3.35 mmol/L ammonia and (B) 97:3 (v/v) chloroform:methanol containing 0.0125% formic acid. Using a flow rate of 0.3 mL/min, the LC gradient was maintained as follows: 10% A from 0 to 1 min; reach 20% A at 4 min; reach 85% A at 12 min; reach 100% A at 12.1 min; 100% A from 12.1 to 14 min; reach 10% A at 14.1 min; 10% A from 14.1 to 15 min. For reversed phase separation, 5 μL of each sample was injected onto a Waters HSS T3 column (150 × 2.1 mm, 1.8 μm particle size). Column temperature was held at 60 °C. The mobile phase consisted of (A) 4:6 (v/v) methanol:water and (B) 1:9 (v/v) methanol:isopropanol, both containing 0.1% formic acid and 10 mmol/L ammonia. Using a flow rate of 0.4 mL/min, the LC gradient was maintained as follows: 100% A at 0 min; reach 80% A at 1 min; reach 0% A at 16 min; 0% A from 16 to 20 min; reach 100% A at 20.1 min, 100% A from 20.1 to 21 min. MS data was acquired by applying negative and positive ionization using continuous scanning over the range of *m*/*z* 150 to *m*/*z* 2000. Data was analyzed using an in-house developed metabolomics pipeline written in the R programming language. Lipid identification is based on a combination of accurate mass, (relative) retention times, fragmentation spectra and injecting relevant standards. Official lipid nomenclature and annotations were used to describe lipid class and species and are listed in Supplemental Table 2. The levels of NADPH and NADP^+^ were measured by the metabolomics core of the Amsterdam UMC [46].

### 2.10 Histological analysis

Liver tissue was fixed in 4% PFA in PBS and embedded in paraffin for H&E staining. For Oil Red O (ORO) staining, tissue was embedded in Tissue Tek (Sakura Finetek) snap-frozen in isopentane at −80 °C. Subsequently, Cryo-sectioning was performed in a cryostat at −18 °C and tissue sections were fixed in 4 % paraformaldehyde for 5 min at RT. Thereafter, slides were washed for 5 min in 60 % isopropanol, and stained with 0.3 % ORO (w/v) solution in 60% isopropanol for 10 min at RT. Next, the tissue slides were washed again in 60 % isopropanol, counterstained for 10 s with hematoxylin, washed 4 times with water and mounted onto cover slips using VectaMount. The tissue slides were imaged using a Leica DM6 B microscope. Neutral lipid accumulation was quantified as the ORO-positive tissue area relative to total tissue area using ImageJ. For ORO staining, 4 × 10^5^ HepG2 cells were seeded in 6-well plates and cultured for 48 h. After washing with PBS, cells were fixed in 4% PFA in PBS for 10 min at room temperature and washed three times with PBS. Samples were incubated in 60% isopropanol for 15 min, followed by at 10 min incubation with 0.3% (w/v) ORO in 60% isopropanol. After rinsing with water, cells were counterstained with hematoxylin and imaged using an EVOS M7000 imaging system (Thermo Fisher). Neutral lipid accumulation was quantified as the ORO-positive area relative to the total cell-covered area using ImageJ.

### 2.11 TG hydrolase activity measurement

Neutral TG hydrolase (TGH) activity in liver tissue was determined at pH 7.0 using a radiolabeled substrate as described previously with minor modifications [47]. Frozen liver samples were homogenized in neutral lysis buffer (100 mM potassium phosphate, 1 mM dithiothreitol; pH 7.0), sonicated twice on ice for 10 s, and centrifuged at 20000×*g* for 10 min at 4 °C. TGH activity was determined in 50 μg of protein from the supernatant. The substrate mixture contained 300 μM triolein per sample supplemented with 0.5 μCi [9,10-^3^H(N)]-triolein (PerkinElmer) and 45 μM mixed phosphatidylcholine/phosphatidylinositol micelles (3:1) in 100 mM potassium phosphate buffer (pH 7.0) and fatty acid-free BSA at a final concentration of 2%. The samples were incubated with the substrate for 1 h at 37 °C, and the reaction was terminated by addition of 3.25 mL stop solution (methanol:chloroform:heptane) (10:9:7, v/v/v) and 1 mL of 100 mM potassium carbonate (pH 10.5). After centrifugation at 322×*g* for 15 min at 4 °C, the radioactivity in 1 mL of the upper phase was determined by liquid scintillation counting (Tri-Carb 2810 TR, PerkinElmer) and neutral TGH activity was expressed as the release of FA/h/mg protein.

### 2.12 Measurement of hepatic de-novo lipogenesis

Hepatic *de novo* lipogenesis (DNL) was measured as we previously reported [38,48]. Briefly, the indicated male littermate mice were given 2% [1-^13^C]acetate (Sigma) in their drinking water for 72 h before sacrifice. Hepatic lipids were extracted and then hydrolyzed and transmethylated by incubation in methanol/HCl (6N) in a 5:1 ratio at 90 °C for 4 h in the presence of 100 μg C17:0 as internal standard and 0.4 mg/mL of butylated hydroxytoluene (BHT). Subsequently, the methylated fatty acids were extracted twice with 2 mL hexane, dried under a stream of nitrogen and resuspended in 200 μL of heptane. Incorporation of ^13^C-acetate into C16:0, C16:1, C18:0, and C18:1 was determined by GC/MS (Agilent 5975C GC/MSD, Agilent Technologies, Santa Clara, CA) using ammonia-induced chemical-ionization (CI). Total hepatic fatty acid concentrations were determined by gas chromatography. Fractional hepatic fatty acid synthesis and chain elongation were calculated using mass isotopomer distribution analysis (MIDA) [49]. Total amounts of fatty acids derived from *de novo* synthesis and chain elongation were calculated by multiplying fractional synthesis rates by the total amounts of the respective fatty acid present in the livers. Newly synthesized cholesterol was determined following hydrolysis of cholesteryl esters in Bligh and Dyer-extracted lipids by incubating them for 16 h at 45 °C in a mixture of 0.2 mL toluene, 1.5 mL methanol and 0.3 mL 8% (m/v) HCl. Subsequently, cholesterol was extracted three times with hexane. The samples were dried under a stream of nitrogen and cholesterol was trimethylsylilated by adding 100 μL pyridine/N,O-Bis(trimethylsilyl) trifluoroacetamide (BSTFA)/Trimethylchlorosilane (TMCS) in a 50:50:1 ratio. Samples were then dried again under nitrogen, taken up in 200 μL heptane, and analyzed by GC/MS to determine isotope incorporation. The fractions of cholesterol that were derived from *de novo* synthesis were calculated using (MIDA) [49]. Total amounts of newly synthesized cholesterol were calculated by multiplying the fractions of newly synthesized cholesterol by the hepatic total cholesterol contents that had been determined using commercially available reagents (Roche).

### 2.13 Immunoblot analysis and co-immunoprecipitation

Liver homogenates were prepared in 1 mL radio-immunoprecipitation assay (RIPA) buffer (Boston Biochem) supplemented with phenylmethylsulfonyl fluoride (Sigma) and protease inhibitors (Roche) using a tissue lyser system (QIAGEN). Lysates were cleared by centrifugation at 4 °C for 10 min at 10,000×*g* and the protein concentration was measured using a BCA assay (ThermoFisher) according to the manufacturer's protocol. Proteins were separate in 4%–12% Bis-Tris NUPAGE gradient gels with MOPS as running buffer (Thermo Fisher Scientific). Thereafter, proteins were transferred unto nitrocellulose membranes, blocked in 5% milk (Elk) in PBS supplemented with 0.5% Tween and blotted with the indicated primary antibodies listed in Supplemental Table 3. This table also indicates all the commercial and self-produced antibodies we used to attempt to detect endogenous MARCHF6. Secondary HRP-conjugated antibodies (Invitrogen) were used for chemiluminescence imaging on a GE IQ800 (GE). Blots shown are representative of at least 3 independent experiments with similar results.

### 2.14 Quantitative PCR

RNA was isolated using Tri reagent (Sigma), cleaned up using the Direct-zol RNA miniprep kit (Zymo Research) and reverse transcribed into cDNA using iScript reverse transcription reagent (BioRad) according to the manufacturer's protocol. Real-time quantitative PCR (qPCR) was performed using SensiFAST SYBR (Bioline) on a LightCycler 480 II system (Roche). Relative gene expression was calculated using the ΔΔCt method and normalized to the expression levels of Rplp0/Rpl13a. Oligonucleotide sequences used for qPCR are provided in Supplemental Table 4.

### 2.15 Statistics

Data are presented as the mean ± standard deviation and statistical analysis was performed using Prism v10 software. Statistical significance was tested using ANOVA with Holm-Šídák post hoc analysis or Welch's t-test with Holm-Šídák correction for multiple comparisons where needed. In case the assumption of normality was violated, Kruskal–Wallis with Dunn's multiple comparison test was used as an alternative to the ANOVA analysis and Mann Whitney test as an alternative to the t-test was employed. Data sets were tested for outliers using Prism's ROUT method with the Q coefficient set to 1%.

## 3 Results

### 3.1 Generation and characterization of global- and hepatocyte-specific MARCHF6 knockout mice

**Figure 1:**
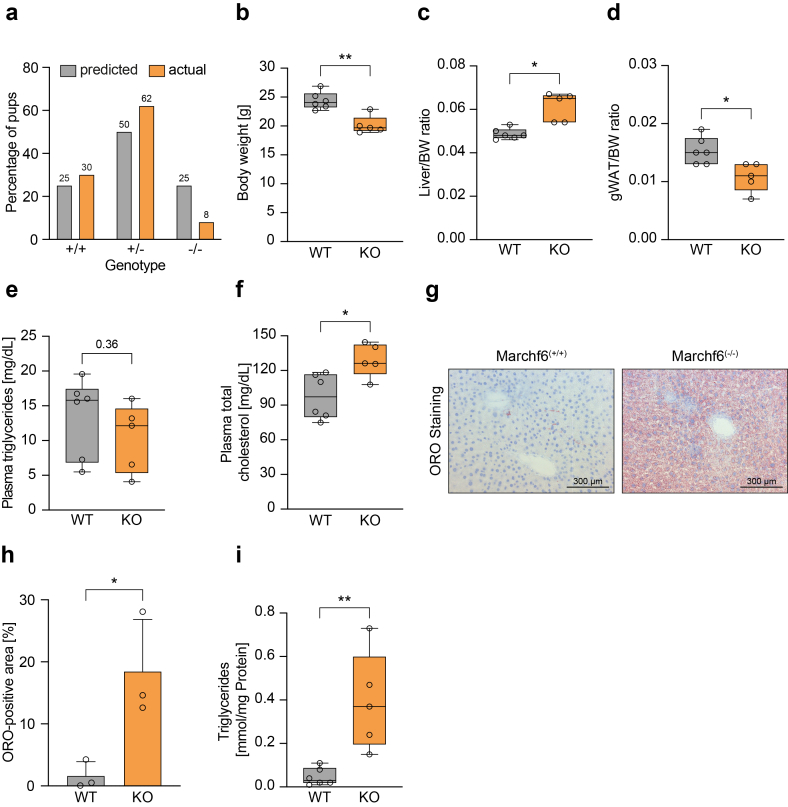
**Characterization of global *Marchf6*^*(−/−)*^ mice.** (**a**) Predicted and actual genotype distribution of 387 pubs born from *Marchf6*^*(+/−)*^ breeding pairs. (**b-i**) Male *Marchf6*^*(−/−)*^ (n = 5) and control littermates (n = 6) were sacrificed at 10 weeks of age and (**b**) bodyweight, (**c**) liver/bodyweight ratio and (**d**) gWAT/bodyweight ratio were determined. (**e**) Plasma triglyceride and (**f**) total cholesterol concentrations were measured. (**g,h**) Hepatic neutral lipids were stained with Oil Red O. (**g**) Representative staining of *Marchf6*^*(−/−)*^ and control littermates and the (**h**) quantification of images from independent mice (n = 3) are shown. (**i**) Hepatic triglyceride content of control and *Marchf6*^*(−/−)*^ mice. Box plots show the median (middle line) 25th, 75th percentile (box) and minimum and maximum values (whiskers). ∗p < 0.05, ∗∗p < 0.01 as analyzed by two-tailed Welch's t-test.

To study the physiological role of *Marchf6,* we generated a mouse model carrying a constitutive deletion of exon 3 (*Marchf6*^*(+/−)*^) and a second one carrying LoxP sites flanking exon 3 (*Marchf6*^*(+/fl)*^) (Supplementary Fig. 1A). Mating of heterozygote *Marchf6*^*(+/−)*^ mice resulted in birth of offspring with a marked deviation from the anticipated Mendelian distribution, most of which did not survive past weaning (Figure 1A). A cohort of surviving male *Marchf6*^*(−/−)*^ mice, collected over an extended period, was significantly lighter and showed growth retardation (Figure 1B and not shown). When normalized to body weight, these mice exhibited a relative larger liver weight and lower gonadal fat mass (Figure 1C,D). We also assessed plasma lipid levels and found that global loss of *Marchf6* did not alter circulating plasma TG but resulted in slightly higher levels of plasma cholesterol compared to wildtype controls (Figure 1E,F). In a small cohort of surviving female *Marchf6*^*(−/−)*^ mice we observed similar growth retardation and a trend to increased relative liver mass (Supplementary Fig. 1B and C). Macroscopic examination of male *Marchf6*^(−/−)^ revealed a pale liver, and histological staining showed marked accumulation of neutral lipids in mice lacking *Marchf6* (Figure 1G,H), which was corroborated by elevated hepatic TG levels (Figure 1I).

**Figure 2:**
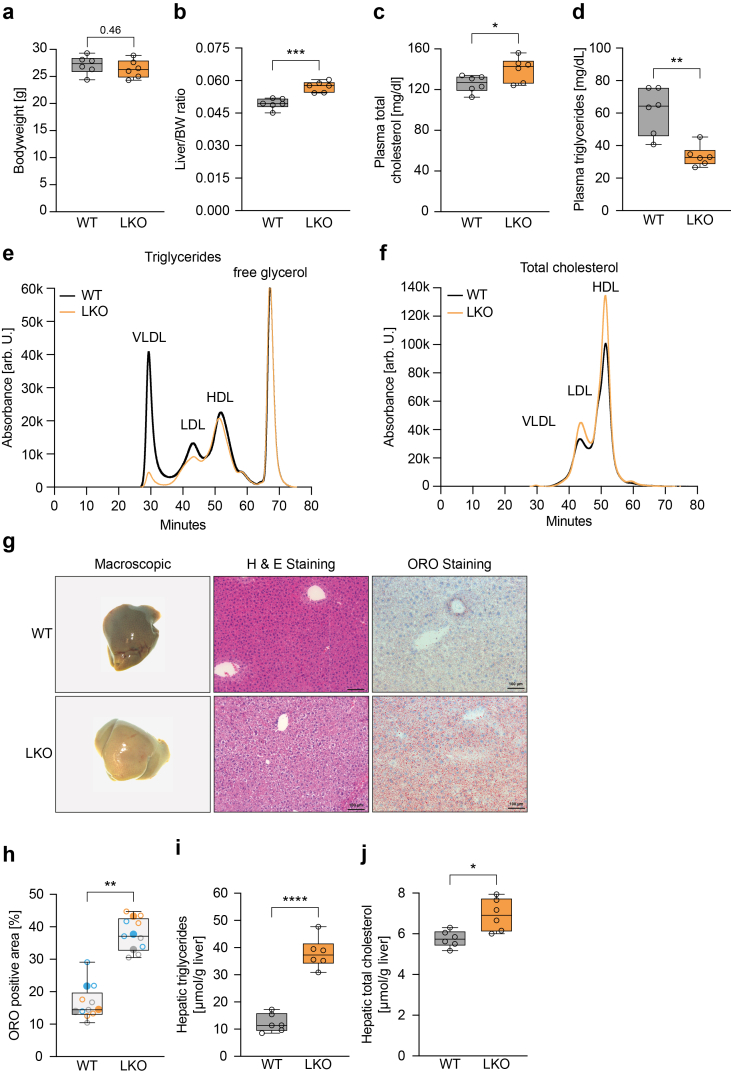
**Hepatic deletion of *Marchf6* alters the plasma lipid profile and leads to hepatic neutral lipid accumulation.** (**a-j**) Male *Marchf6*^*LKO*^ (n = 6) and control littermates (n = 6) on standard chow diet were sacrificed at 12 weeks of age and (**a**) bodyweight, (**b**) liver/bodyweight ratio, (**c**) plasma triglyceride and (**d**) total cholesterol levels were determined. (**e,f**) Plasma of mice from the same genotype (WT, LKO, n = 6) was pooled, size fractionated and triglyceride and total cholesterol content of the lipoprotein fractions was determined. (**g**) Representative macroscopic, H&E and Oil Red O-stained liver images from WT and LKO liver sections. (**h**) Quantification of neutral lipids amounts of liver tissue sections stained with Oil Red O. For each group, images (N = 3) from 3 independent mice were analyzed. Measurements are color coded per mouse, and the individual values (empty symbols) and average (full symbol) are shown. Hepatic (**i**) triglyceride and (**j**) total cholesterol content of WT and LKO mice. Box plots show the median (middle line) 25th, 75th percentile (box) and minimum and maximum values (whiskers). ∗p < 0.05, ∗∗p < 0.01, ∗∗∗p < 0.001 and ∗∗∗∗p < 0.0001 as analyzed by two-tailed Welch's t-test.

Due to the skewed Mendelian birth ratio of *Marchf6*^*(−/−)*^ mice and the liver being a main hub of lipid metabolism, we developed a mouse model lacking hepatocyte *Marchf6 (Marchf6*^*LKO*^*)* expression (Supplementary Figs. 1A and 2A)*. Marchf6*^*LKO*^ mice were born at the expected Mendelian ratio (not shown) and after 12 weeks on standard chow diet had a similar body weight as their control counterparts (Figure 2A), but their livers were enlarged (Figure 2B and Supplementary Fig. 2B). Analysis of plasma lipids revealed that loss of hepatocyte *Marchf6* resulted in significant lower circulating TG levels, evident in the VLDL and LDL lipoprotein fractions, and slightly elevated circulating cholesterol levels (Figure 2C–F). Unlike plasma lipid levels, fasting plasma glucose levels and glucose tolerance were unchanged in *Marchf6*^LKO^ mice (Supplementary Fig. 2C and D). To test whether loss of hepatocyte *Marchf6* causes liver damage, plasma transaminase levels were measured. Both aspartate aminotransferase (AST) and alanine transaminase (ALT) levels were comparable in *Marchf6*^LKO^ and WT mice, indicating that liver-specific deletion of *Marchf6* is not hepatotoxic (Supplementary Fig. 2E). Macroscopic examination of *Marchf6*^LKO^ livers revealed visible fat accumulation, which was corroborated by histological analysis showing spatially dispersed LDs rich in neutral lipids (Figure 2G,H). Hepatic lipid accumulation in *Marchf6*^LKO^ mice was characterized by a pronounced increase in hepatic TGs, accompanied by a moderate increase in total cholesterol content (Figure 2I,J).

### 3.2 Loss of MARCHF6 reprograms hepatic lipidome metabolism

**Figure 3:**
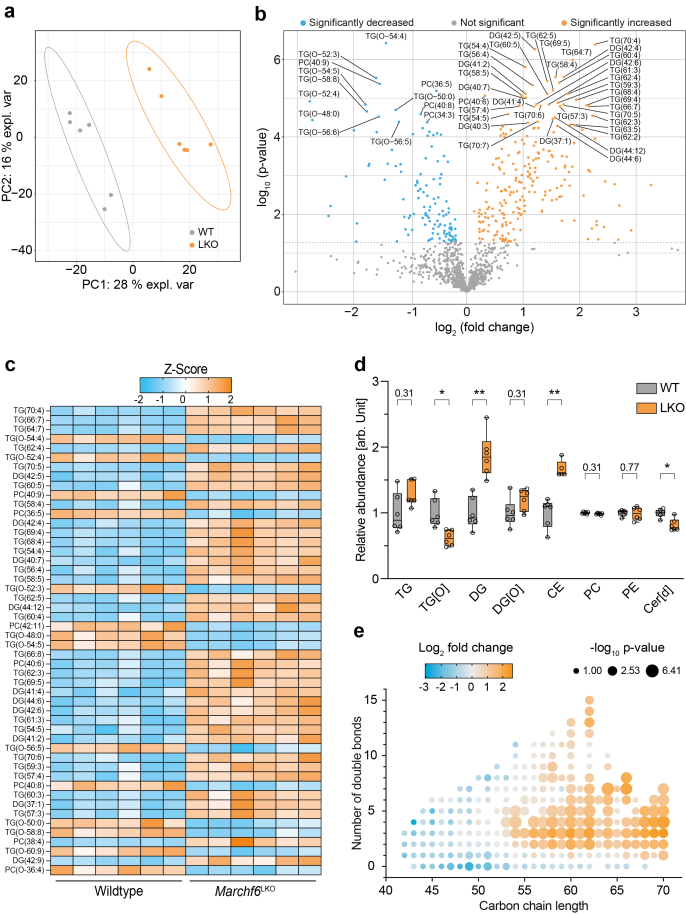
**Loss of *Marchf6* remodels the hepatic lipidome.** (**a-e**) 10-week-old chow-fed male WT (n = 6) and *Marchf6*^*LKO*^ (n = 6) mice were fasted for 4 h, sacrificed, and liver tissue was subjected to lipidomic profiling. (**a**) Principal component analysis of lipidomic data from livers of WT and LKO mice. (**b**) Volcano blot with each dot representing one of the 1151 lipids identified. Orange coloring indicates a significant increase whereas blue coloring indicates a significant decrease. The 50 most changed lipid species (LKO vs. WT) based on p-value are labeled. The horizontal dotted line indicates a p-value of 0.05. (**c**) Heatmap showing the 50 most changed lipids (LKO vs. WT) sorted on their Variable Importance Projection (VIP) score [62] (**d**) Relative abundance of a selection of lipid species. (**e**) Correlation matrix of carbon chain length and number of double bonds in all measured triglyceride species. Color represents the log_2_ fold change (LKO vs. WT) and dot size represents the -log10 p-value as indicated in the legend. (**d**) Box plots show the median (middle line) 25th, 75th percentile (box) and minimum and maximum values (whiskers). ∗p < 0.05, ∗∗p < 0.01, as analyzed by unpaired, two-tailed Welch's t-test with Holm-Šídák correction for multiple comparison.

To gain a more detailed insight into the effects of *Marchf6* deletion on the hepatic lipid profile, we performed a lipidomic analysis. Principal component analysis showed a clear separation of samples derived from control and *Marchf6*^LKO^ livers suggesting a considerable influence of *Marchf6* on the overall hepatic lipid profile (Figure 3A). In the absence of MARCHF6, hepatic lipids are markedly remodeled, with increased di- and tri-TGs and cholesteryl esters alongside decreased phosphatidylcholine, alkyl-diacylglycerols, and ceramide species suggesting a disrupted balance between neutral lipid storage and membrane/signaling lipids (Figure 3B–D). The accompanying shift toward longer, more unsaturated fatty acids points towards changes in the fatty acid elongation and desaturation molecular machinery, likely driven by MARCHF6-regulated metabolic and transcriptional pathways (Figure 3E). Collectively, these findings identify MARCHF6 as a key regulator of hepatic lipid homeostasis. Its loss triggers a coordinated lipidome shift that enhances neutral lipid storage and promotes the accumulation of longer, more unsaturated fatty acids characteristic of hepatosteatosis.

**Figure 4:**
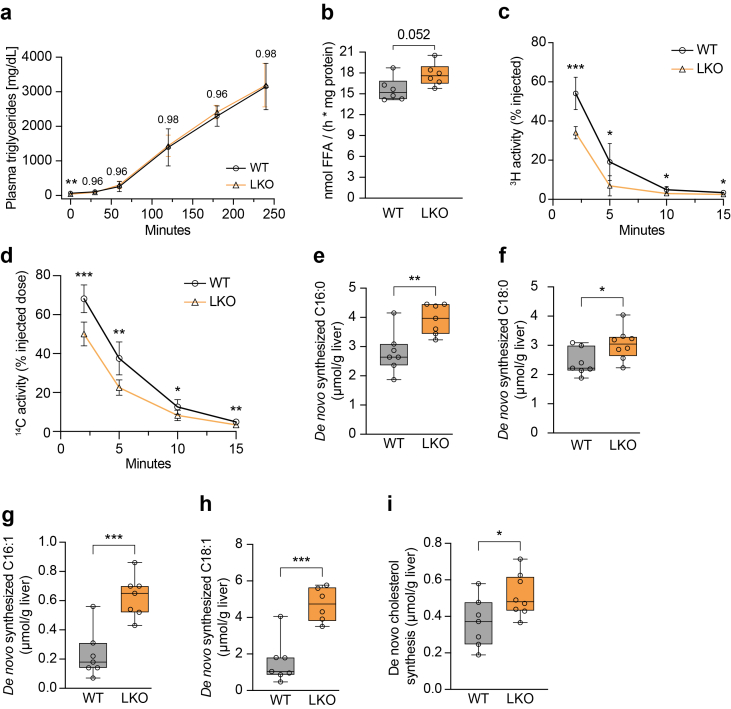
**Ablation of *Marchf6* increases hepatic lipoprotein uptake and *de novo* lipogenesis**. (**a**) 10-week-old chow-fed male WT and *Marchf6*^*LKO*^ (n = 5/group) mice were injected intraperitoneally with Poloxamer 470 (1 g/kg) to block peripheral LPL activity. Plasma was collected at the indicated time points and triglyceride content was measured. (**b**) Liver protein lysates of 10-week-old male WT and *Marchf6*^*LKO*^ (n = 6/group) mice were incubated with substrate containing [^3^H]triolein and the release of free fatty acids was measured by liquid scintillation counting. (**c,d**) 15-week-old male WT and *Marchf6*^*LKO*^ (n = 8/group) were injected with [^3^H]triolein- and [^14^C]cholesteryl oleate-labeled TG-rich lipoprotein-like particles. Plasma was drawn via the tail vein at the indicated timepoints and (**c**) ^3^H and (**d**) ^14^C radioactivity measured by liquid scintillation counting. (**e**–**i**) 10-week-old male WT and *Marchf6*^*LKO*^ (n = 6/group) were given ad libitum access to water containing 2% [^13^C]acetate for three days. Subsequently, livers were collected and the incorporation of [^13^C]acetate into the indicated lipid species was measured. (**a**,**c,d**) Each data point represents the mean value, error bars represent the SD. (**b,e-i**) Box plots show the median (middle line) 25th, 75th percentile (box) and minimum and maximum values (whiskers). ∗p < 0.05, ∗∗p < 0.01 and ∗∗∗p < 0.001 as analyzed by unpaired, two-tailed Welch's t-test with (**a**–**c**) Holm-Šídák correction for multiple comparison.

Given the strong effect of hepatocyte loss of *Marchf6* on TG homeostasis we sought to identify the underlying processes. To test whether VLDL secretion is impaired, we inhibited peripheral LPL activity in fasted mice and measured the accumulation of circulating TG over time, a proxy for hepatic VLDL-TG secretion. No difference in plasma TG levels was observed between control and *Marchf6*^LKO^ mice upon LPL inhibition, indicating intact VLDL-mediated lipid export in the absence of MARCHF6 (Figure 4A). Additionally, neutral TG hydrolase activity was comparable in WT and *Marchf6*^LKO^ liver samples, suggesting that TG mobilization from intrahepatic LDs is functional and not impaired (Figure 4B). Alternatively, increased hepatic lipid content in LKO mice may reflect enhanced lipoprotein-derived lipid uptake. To test this notion, mice were injected with glycerol tri[^3^H]triolein and [^14^C]cholesteryl-oleate recombinant VLDL-like particles and plasma clearance and tissue uptake were followed over time. Plasma clearance of the radiolabeled particles was rapid in the control WT mice, and already at the first time point measured (2 min) a substantial fraction of the injected tracer was cleared. In the absence of hepatocyte MARCHF6, tracer clearance from the plasma was significantly accelerated (Figure 4C,D). Rapid plasma clearance in genotypes was mirrored by accumulation of radioactivity in tissues, with [^14^C]cholesterol predominantly being taken up in the liver, heart and spleen, and glycerol tri[^3^H]oleate-derived activity accumulating in the liver, heart, perivascular fat, and spleen (Supplementary Fig. 3A and B). Although the difference in tissular accumulation of radioactivity did not reach statistical significance, a trend toward increased hepatic uptake of both tracers was observed in *Marchf6*^LKO^ mice. Combined with the markedly faster plasma clearance of the tracers in *Marchf6*^LKO^ mice, this may contribute, at least in part, to the hepatic lipid accumulation observed in these mice.

As shown in Figure 3E, the length and desaturation index of TG-incorporated fatty acids were increased in livers of *Marchf6*^LKO^ mice. This may point towards increased synthesis, elongation, and desaturation of fatty acids in the absence of MARCHF6. To test this idea, mice were given [^13^C]acetate in their drinking water for 3 days and the fractional incorporation of this substrate into *de novo* synthesized fatty acids and cholesterol was evaluated. We found a marked increase in hepatic synthesis of C16:0 and C18:0 fatty acids and desaturation (C16:1 and C18:1, respectively) in *Marchf6*^LKO^ mice (Figure 4E–H). Likewise, cholesterol synthesis was increased in the livers of *Marchf6*^LKO^ mice, which is consistent with MARCHF6 being an established regulator of the rate-limiting enzymes HMGCR and SQLE of cholesterol biosynthesis (Figure 4I). We also considered the possibility that *Marchf6*^*LKO*^ mice change their feeding behavior or shift to different substrates for energy production and performed indirect calorimetry. Food and water intake were comparable between WT and *Marchf6*^LKO^ mice (Supplementary Fig. 4A and B). LKO mice did not display neither increased fat nor carbohydrate oxidation, or elevated total energy expenditure (Supplementary Fig. 4C–E). Similarly, the balance between O_2_ consumption and CO_2_ production, and therefore the respiratory exchange ratio (RER) was unchanged in the absence of hepatocyte MARCHF6 (Supplementary Fig. 4F–H). These findings indicate that food intake and energy substrate utilization are unlikely to contribute to the lipid phenotype observed in *Marchf6*^LKO^ mice. In aggregate, our results point towards enhanced hepatic lipid uptake and synthesis as a primary cause for lipid accumulation in the absence of MARCHF6.

### 3.3 Loss of hepatocyte MARCHF6 promotes SREBP1-associated lipogenesis

**Figure 5:**
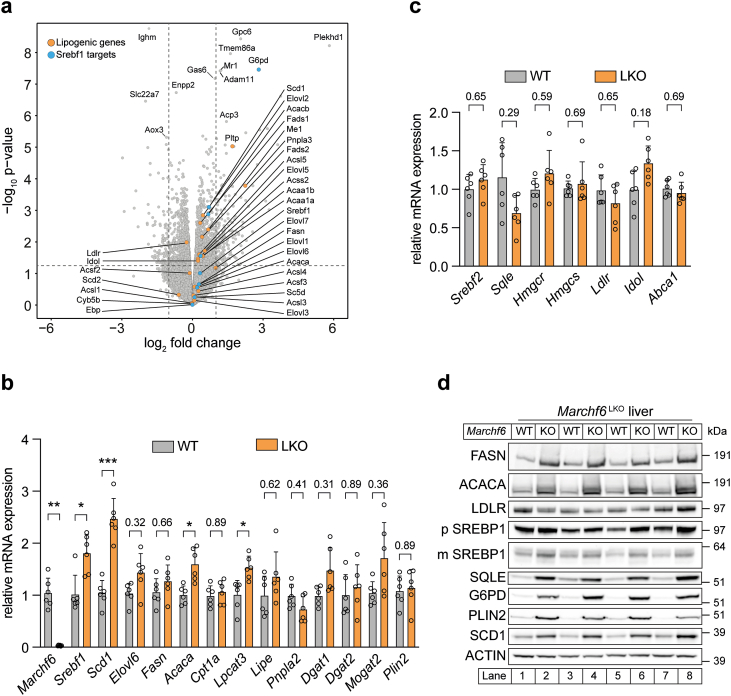
**Increased lipogenic gene program in LKO mice.** (**a**) Livers of chow-fed male WT and *Marchf6*^*LKO*^ (n = 6/group) mice were transcriptionally profiled. Log_2_ fold changes in gene expression (LKO vs. WT) are represented in a volcano plot. Vertical dotted line represents a fold change of ±1 and horizontal dotted line indicates a p-value of 0.05. (**b,c**) Expression of the indicated genes was assessed by qPCR in livers of WT and *Marchf6*^*LKO*^ (n = 6/group) mice. (**d**) Liver lysates from the same mice as in (**a)** were immunoblotted for the proteins indicated. (**b,c**) Bars represent the mean value and the error bars represent SD. ∗p < 0.05, ∗∗p < 0.01 and ∗∗∗p < 0.001 as analyzed by unpaired, two-tailed Welch's t-test with Holm-Šídák correction for multiple comparison.

To investigate the mechanisms underlying the observed increase in *de novo* lipid synthesis, we transcriptionally profiled livers of WT and *Marchf6*^*LKO*^ mice. Bulk mRNA sequencing of *Marchf6*^LKO^ liver tissue did not reveal obvious affected pathway(s) that could explain the lipid accumulation (Supplementary Fig. 5A). We therefore analyzed the data in more detail and checked whether known lipogenic genes were differentially expressed in *Marchf6*^LKO^ livers compared to their wildtype littermates and identified a modest overall upregulation of SREBP1c-regulated lipogenic genes (Figure 5A). This observation was confirmed by direct measurement of a selection of SREBP1-target genes, while genes associated with TG catabolism and LD formation were unaffected by the loss of hepatic *Marchf6* (Figure 5B), which is also consistent with unchanged TGH activity (Figure 4B). A similar SREBP1c-associated gene expression profile was observed in livers of global *Marchf6*^(−/−)^ mice (Supplementary Fig. 5B). Distinct from SREBP1 target genes, SREBP2-regulated genes associated with cholesterol biosynthesis were not affected by the ablation of *Marchf6* in the liver (Figure 5C). Given that the MARCHF6 is a an ERAD-associated E3 ubiquitin ligase that primarily regulates cellular processes at the post-translational level, we expected a more pronounced effect on protein abundance. Consistent with this idea, levels of SREBP1 precursor and mature form and its downstream targets (*e.g.* ACACA, FASN, SCD1, G6PD) were markedly increased in *Marchf6*^LKO^ and *Marchf6*^(−/−)^ livers. In addition, SQLE and PLIN2, two established MARCHF6 targets and proxies for its activity [22,31], were more abundant in those mice (Figure 5D and Supplementary Fig. 5C). Despite extensive efforts with multiple commercial and self-raised antibodies we were unable to detect endogenous MARCHF6 by immunoblotting (Supplemental Table 3). Of note, hepatic LDLR abundance was unchanged in the absence in *Marchf6*^LKO^, which is different from what was previously reported in hepatoma cell lines as a consequence of concomitant *IDOL* induction [30]. The most parsimonious explanation for the enhanced activation of the SREBP1 pathway is that SREBP1 itself, whose precursor and mature form are increased in the absence of MARCHF6, is a direct ubiquitination and degradation target of MARCHF6. However, our initial experiments do not seem to support this scenario (not shown), leaving the mechanism underpinning this observation unresolved.

### 3.4 Cell autonomous lipid accumulation in murine and human hepatocytes lacking MARCHF6

**Figure 6:**
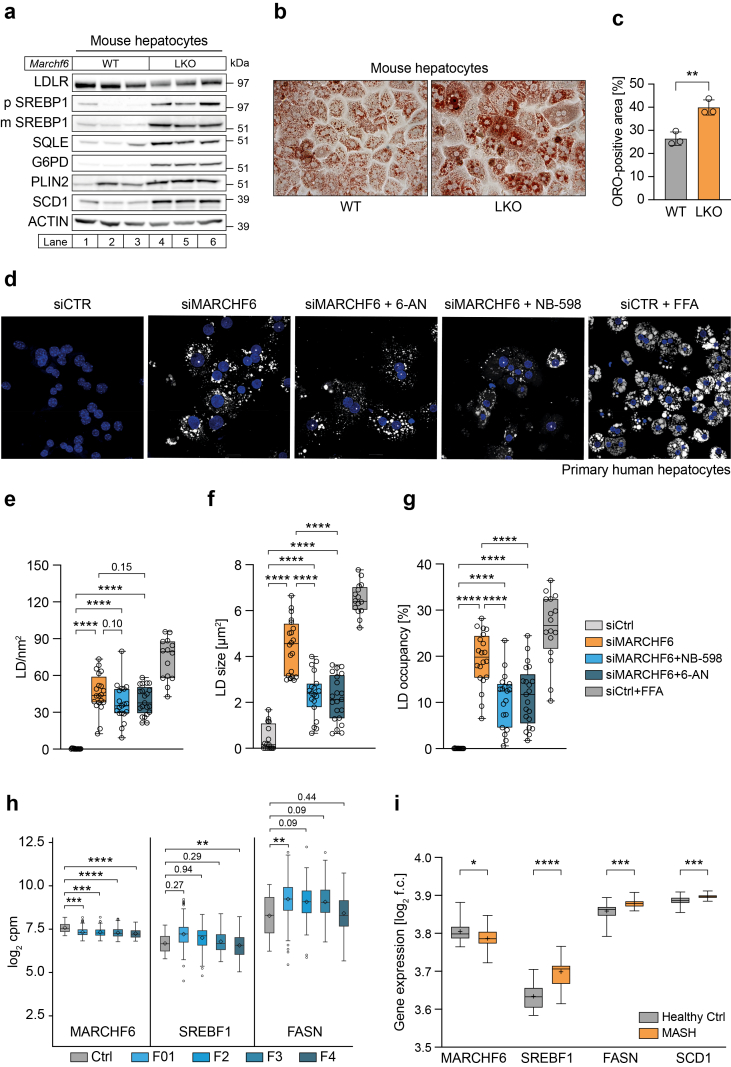
**Decreased MARCHF6 activity results in cell-autonomous lipid accumulation**. (**a**) Primary mouse hepatocytes were isolated from livers of male WT and *Marchf6*^*LKO*^ mice (n = 3/group), lysed and immunoblotted for the indicated proteins. (**b,c**) ORO staining of isolated primary mouse hepatocytes after 24 h in culture (n = 3/group). (**b**) Representative images of stained WT and LKO hepatocytes and (**c**) the quantification of the ORO-positive area is shown. (**d-g**) Primary human hepatocytes were transfected with Ctrl or *MARCHF6* siRNAs and subsequently treated with 6-AN (pentose phosphate pathway inhibitor), NB-598 (squalene epoxidase inhibitor) or free fatty acids (positive control) for 24 h. (**d**) Representative confocal microscopy images of treated primary human hepatocytes stained with BODIPY to visualize LDs. DAPI was used to stain nuclei. (**e**–**g**) Quantification of (**e**) LD number, (**f**) LD size and (**g**) LD occupancy. 16–21 individual cells from 3 independent experiments were analyzed per treatment condition. (**h**) Hepatic gene expression of *MARCHF6*, *SREBF1* and *FASN* in MAFLD patients, extracted from the SteatoSITE transcriptome browser (total of 632 MASLD spectrum patients and 32 controls; https://shiny.igc.ed.ac.uk/SteatoSITE_gene_explorer/). Patients were categorized in 5 groups (Ctrl -F4) based on fibrosis score. (**i**) Comparison of hepatic *MARCHF6*, *SREBF1*, *FASN,* and *SCD1* expression between healthy control (n = 24) and MASH patients (n = 19). Data was extracted from Arendt et al. [51] using the R2 Genomics Analysis and Visualization Platform (http://r2platform.com). (**e-g**, **h,i**) Box plots show the median (middle line) 25th, 75th percentile (box), minimum and maximum values (whiskers) and (h,**i**) mean values are indicated by a ◊ and +, respectively. ∗p < 0.05, ∗∗p < 0.01, ∗∗∗p < 0.001 and ∗∗∗∗p < 0.0001 as analyzed by ordinary one-way ANOVA with Holm-Šídák post hoc analysis to correct for multiple comparisons.

To assess whether our observations in liver are cell autonomous, we analyzed the abundance of proteins involved in lipogenesis in isolated primary hepatocytes from *Marchf6*^LKO^ mice and in HepG2 cells with MARCHF6 deletion (Supplementary Fig. 6A). Like in intact liver, protein levels of both the precursor and mature forms of SREBP1 protein as well as that of SREBP1 downstream targets *e.g.* SCD1 and G6PD were increased, which was also the case for the two *bona fide* MARCHF6 targets SQLE and PLIN2 (Figure 6A and Supplementary Fig. 6B). Furthermore, ORO staining showed that absence of MARCHF6 led to the accumulation of neutral lipids in primary mouse hepatocytes and HepG2 cells, comparable to what was observed in liver tissue sections (Figure 6B,C and Supplementary Fig. 6C and D). We next examined the effect of *MARCHF6* silencing in primary human hepatocytes (PHH). In line with our findings in mouse livers and HepG2 cells, expression of SREBP1 and its lipogenic target genes was increased in PHH upon *MARCHF6* silencing (Supplementary Fig. 6E). The mRNA expression of DGAT1, an enzyme involved in TG synthesis, was only marginally elevated, while expression of DGAT2 was unchanged (Supplementary Fig. 6E). Accordingly, silencing of *MARCHF6* led to neutral lipid accumulation, as demonstrated by staining with the lipophilic fluorophore BODIPY (Figure 6D). Morphometric analysis of LDs in *MARCHF6*-silenced PHH revealed that these cells contained more LDs and that the droplets were bigger, resulting in a larger fraction of the intracellular space being occupied by LDs compared to control cells (Figure 6E–G). As increased SQLE abundance is a common consequence of reduced MARCHF6 activity, we asked whether its inhibition affects the LD phenotype in PHH. Treatment of *MARCHF6-*silenced PHHs with the high affinity SQLE inhibitor, NB-598, markedly reduced LD size and occupancy, presumably due to limiting *de novo* cholesterol synthesis. Additionally, MARCHF6-regulated lipogenic enzymes require NADPH for their activity [28]. Therefore, we questioned whether treatment with 6-aminonicotinamide (6-AN), an antimetabolite that blocks the NADPH-producing pentose phosphate pathway, would counter lipid accumulation in *MARCHF6-*silenced PHH. Indeed, 6-AN treatment significantly reduced LD size and occupancy in PHHs (Figure 6F,G). This result further supports NADPH-dependent *de novo* lipogenesis as an important driver of lipid accumulation upon MARCHF6 deficiency, yet at this point we cannot rule out other 6-AN-induced pleiotropic effects. Interestingly, in livers of *Marchf6*^LKO^, despite elevated mRNA and protein levels of G6PD, the rate limiting enzyme of the NADPH-producing pentose phosphate pathway (Figure 5, Figure 6A and Supplementary Fig. 5B and C), the NADP+/NADPH ratio was significantly increased compared to WT livers (Supplementary Fig. 6F). This may suggest that loss of hepatocyte MARCHF6 results in excessive lipid synthesis, potentially depleting hepatic NADPH reserves. Taken together these findings in human primary hepatocytes reinforce our observations in the mouse model, demonstrating that the lipid accumulation observed in cells with diminished *MARCHF6* expression results, at least in part, from increased *de novo* lipogenesis.

One feature shared between metabolic dysfunction-associated steatotic liver disease (MASLD) and steatohepatitis (MASH) is accumulation of lipids in the liver. As this feature is observed in the *Marchf6*^*LKO*^ mouse model, we aimed to gain initial insight into the potential role of MARCHF6 in these pathologies. To this end, we analyzed publicly available transcriptomic data from SteatoSITE [50] and Arendt et al. [51], which contain expression profiles from MASLD and MASH patients, respectively. Both datasets revealed that MARCHF6 expression decreased with disease progression, while expression of *SREBF1* and its target genes *FASN* and *SCD1* was moderately upregulated (Figure 6H,I). This is consistent with our observations in the *Marchf6*^*LKO*^ mouse model and highlights the potential role of MARCHF6 in human steatotic liver disease.

## 4 Discussion

Our study establishes MARCHF6 as a pivotal regulator of hepatic lipid metabolism by demonstrating that its global and hepatocyte-specific loss precipitates marked TG accumulation and steatosis, in the absence of caloric overload or apparent metabolic stress. This phenotype reflects a hepatocyte-autonomous defect rather than a consequence of systemic metabolic dysregulation. Mechanistically, MARCHF6 sits at the interface between lipid synthesis, hepatic lipid clearance, and redox homeostasis. Loss of MARCHF6 disrupts this intricate balance, resulting in hepatic neutral-lipid accumulation and hence may contribute to development of MASLD.

MARCHF6 is an ERAD-associated multivalent E3 ubiquitin ligase which has been implicated it the degradation of multiple substrates and in the N-end rule that controls the half-life of proteins [52,53]. In line with it having an important physiological role, global ablation of *Marchf6* in mice results in a marked shift from the expected Mendelian birth distribution (this study and [29]). In lipid metabolism, MARCHF6 was shown to regulate the proteasomal degradation of the cholesterol biosynthesis enzymes SQLE, DHCR24, LDM, SC4MOL and SC5D [22,23,[25], [26], [27]], as well as of the LD-associated protein PLIN2 [31]. As such, a prominent feature of MARCHF6 loss in hepatocytes is increased abundance of SQLE protein and heightened sterol pathway throughput, as was also observed in a non-hepatic cell system [22]. This increase is also observed *in vivo* in mouse liver and results in elevated, albeit to a more limited extent, hepatic cholesterol production. A striking observation in LKO mice was the development of spontaneous buildup of neutral lipids in LDs, which we attribute to the combination of cholesteryl-ester and acylglycerol accumulation, increased hepatic *de novo* FA synthesis and accelerated hepatic lipoprotein uptake. Importantly, loss of MARCHF6 in human primary hepatocytes recapitulates this phenotype, supporting the translational value of our findings in mice.

The activation of SREBP1 and SREBP2 share a common proteolytic maturation cycle [9], and activation of the SREBP1 pathway necessitates SREBP2-driven cholesterol biosynthesis [54]. This raises a pertinent question: why is the SREBP1-mediated lipogenic axis so robustly activated upon MARCHF6 deficiency, while that controlled by SREBP2 is less affected? Several ERAD-associated E3s have been implicated in the degradation of SREBPs [[16], [17], [18], [19]] and hence the simplest explanation for this discrepancy would be that SREBP1c itself is a direct degradation target of MARCHF6. This could explain why both the precursor and mature (*i.e.* nuclear) forms of SREBP1 are elevated upon MARCHF6 ablation, and that the hepatic phenotype of LKO largely phenocopies that of liver-specific transgenic mice that produce a constitutively-active SREBP1c form [55]. However, our initial results do not support this scenario (not shown). Alternatively, specific lipid species in the ER membrane or the degree of ER membrane desaturation are strong determinants of hepatic SREBP1c activation. Rong et al. [56] have recently shown that elevated ER membrane phosphatidylethanolamine content, following DGAT2-inhibition, attenuates SREBP1c activation. Additionally, studies have demonstrated that hepatic LPCAT3-mediated incorporation of polyunsaturated FA into phospholipids increases the ER membrane desaturation and concomitantly promotes SREBP1c activation [57]. Reciprocally, loss of hepatic LPCAT3, which reduces ER membrane desaturation, has the opposite outcome on SREBP1c signaling. In our study, we observed that loss of hepatocyte MARCHF6 leads to enhanced *in vivo* DNL, FA synthesis, elongation, and desaturation along with SREBP1c activation. A limitation of our study is that the lipidomic profile was obtained from bulk liver tissue and that we did not specifically isolate and evaluate ER membrane fractions. It is nevertheless interesting to speculate that enhanced ER membrane desaturation may contribute to enhanced SREBP1 activation in the absence of MARCHF6.

Our study supports the notion that increased DNL is a prominent determinant of the LD phenotype in livers of LKO mice and this is consistent with the phenotype observed in human hepatocytes, where pharmacological interventions targeting sterol synthesis and NADPH availability reduce LD abundance. As an anabolic process, DNL has a high demand for NADPH as a reducing agent. In line with this heightened NADPH demand, global- and hepatocyte-specific loss of MARCHF6 in mice resulted in elevated abundance of G6PD, the rate-limiting enzyme in the NADPH-producing pentose-phosphate pathway, and an SREBP1-sensitive gene [58,59]. However, despite this increase, the NADP^+^/NADPH ratio was unexpectedly elevated in the livers of LKO mice, suggesting enhanced NADPH consumption in the absence of MARCHF6. Intriguingly, hepatocyte-specific SQLE transgenic mice which, similar to *MARCHF6*^*LKO*^ mice, have increased SQLE protein abundance developed hepatosteatosis and had a similar increase in the NADP^+^/NADPH ratio [60]. MARCHF6 was recently implicated as a direct sensor of cellular NADPH availability, owing to the presence of the C-terminal NADPH-binding MARA domain of MARCHF6 [29]. This study suggested that high NADPH levels promote MARCHF6 ubiquitylation and ERAD of its targets *e.g.* SQLE, PLIN2, and MARCHF6 itself, and that reciprocally, low NADPH limits MARCHF6 activity and promotes their stability. From the perspective of lipid metabolism this seems counterintuitive; when NADPH availability is not limited (*e.g.* high energy state), NADPH-consuming DNL should be supported. In this context, it is intriguing that Nguyen et al. [29] demonstrated that by sensing NADPH, MARCHF6 protects cells from ferroptosis by degrading pro-ferroptotic ASCL4 and P53, as well as the established target SQLE. Conversely, loss of MARCHF6, increased their level and enhanced ferroptosis in an NADPH-dependent manner *in vitro*. This was also associated with a decrease in the anti-ferroptotic SLC7A11, GPX4, and NRF2, which is likely a secondary effect, as it cannot be directly explained by the loss of E3 ligase activity. This finding may suggest a model in which MARCHF6 concomitantly attenuates DNL and ferroptosis in an NADPH-dependent manner [29]. However, in a physiological *in vivo* setting, where loss of MARCHF6 promotes hepatic DNL, we observed strong induction of anti-oxidant and anti-ferroptotic markers (data not shown). We argue that, in addition to promoting lipid storage in LDs, this mechanism serves to protect hepatocytes from lipid peroxidation-associated toxicity in cells with a high DNL capacity. Clearly, the relationship between MARCHF6 and ferroptosis warrants further investigation.

Finally, we provide transcriptional evidence that MARCHF6 expression decreases along the MASLD to MASH progression, accompanied by an increase in SREBP1 targets. The link between DNL and ferroptosis, which are both regulated by MARCHF6, is emerging as a determinant of human MASLD [61]. Moving forward, it will be important to dissect how MARCHF6 activity is regulated in hepatocytes, how it interfaces with DNL and ferroptosis, and whether MARCHF6 dysfunction contributes directly to human MASLD/MASH. Addressing these questions may uncover novel intervention targets for steatotic liver disease and related disorders.

## CRediT authorship contribution statement

**Vinay Sachdev:** Writing – review & editing, Writing – original draft, Visualization, Validation, Methodology, Investigation, Formal analysis, Data curation, Conceptualization. **Nienke M. van Loon:** Writing – review & editing, Writing – original draft, Visualization, Methodology, Investigation, Formal analysis, Data curation, Conceptualization. **Jenina Kingma:** Writing – review & editing, Methodology, Investigation, Formal analysis, Data curation. **Roelof Ottenhoff:** Writing – review & editing, Methodology, Investigation, Formal analysis, Data curation. **Josephine M.E. Tan:** Writing – review & editing, Methodology, Investigation, Formal analysis, Data curation. **Marlene van den Berg:** Writing – review & editing, Methodology, Investigation, Formal analysis, Data curation. **Suzanne Duijst:** Writing – review & editing, Methodology, Investigation, Formal analysis, Data curation. **Aldo Jongejan:** Writing – review & editing, Visualization, Methodology, Investigation, Formal analysis, Data curation. **Johannes H.M. Levels:** Writing – review & editing, Methodology, Investigation, Formal analysis, Data curation. **Patrick C.N. Rensen:** Writing – review & editing, Methodology, Investigation, Formal analysis, Data curation. **Sander Kooijman:** Writing – review & editing, Visualization, Methodology, Investigation, Formal analysis, Data curation. **Jan-Freark de Boer:** Writing – review & editing, Methodology, Investigation, Formal analysis, Data curation. **Folkert Kuipers:** Writing – review & editing, Methodology, Investigation, Funding acquisition, Formal analysis, Data curation. **Katharina B. Kuentzel:** Writing – review & editing, Methodology, Investigation, Formal analysis, Data curation. **Dagmar Kratky:** Writing – review & editing, Methodology, Investigation, Formal analysis, Data curation. **Yun Kwon:** Writing – review & editing, Visualization, Methodology, Investigation, Formal analysis, Data curation. **Anja Zeigerer:** Writing – review & editing, Methodology, Investigation, Formal analysis, Data curation, Conceptualization. **Sebastian Hendrix:** Writing – review & editing, Writing – original draft, Visualization, Validation, Methodology, Investigation, Formal analysis, Data curation, Conceptualization. **Noam Zelcer:** Writing – review & editing, Writing – original draft, Visualization, Validation, Supervision, Project administration, Methodology, Investigation, Funding acquisition, Formal analysis, Data curation, Conceptualization.

## Declaration of generative AI and AI-assisted technologies in the manuscript preparation process

During the preparation of this work the author(s) used Perplexity to synthesize complex literature and to provide an overview of a field or research question. After using this tool/service, the author(s) reviewed and edited the content as needed and take(s) full responsibility for the content of the published article.

## Declaration of competing interest

The authors declare the following financial interests/personal relationships which may be considered as potential competing interests: Noam Zelcer reports financial support was provided by Dutch Research Council. Dagmar Kratky reports financial support was provided by Austrian Academy of Sciences. Folkert Kuipers reports financial support was provided by Noaber Foundation. If there are other authors, they declare that they have no known competing financial interests or personal relationships that could have appeared to influence the work reported in this paper.

## Data Availability

The raw RNAseq data are deposited in the Gene Expression Omnibus database under accession GSE324273. All other data supporting the findings of this study are available within the paper and its Supplementary Information.
