# Supplementary material for: Loss of the E3 ubiquitin ligase MARCHF6 alters hepatic lipid metabolism and drives spontaneous hepatosteatosis
Source: Mol Metab. 2026 May 2;108:102379. doi: 10.1016/j.molmet.2026.102379 (PMC13194171; doi:10.1016/j.molmet.2026.102379)
Supplement: Multimedia component 1 [file mmc1.pdf]

Loss of the E3 ubiquitin ligase MARCHF6 alters hepatic lipid metabolism and drives spontaneous hepatosteatosis

Vinay Sachdev[1,2,3,#], Nienke M. van Loon[1,2,3,#], Jenina Kingma[1,2,3], Roelof Ottenhoff[1,2,3], Josephine M.E. Tan[1,2,3,4], Marlene van den Berg[1,2,3], Suzanne Duijst[5], Aldo Jongejan[6], Johannes H.M. Levels[7], Patrick C.N. Rensen[8], Sander Kooijman[8], Jan-Freark de Boer[9,10], Folkert Kuipers[10,11], Katharina B. Kuentzel[12,&], Dagmar Kratky[12,13], Yun Kwon[14], Anja Zeigerer[14,15,16,17], Sebastian Hendrix[1,2,3, ‡], Noam Zelcer[1,2,3,‡]

Supplemental information

[1] Department of Medical Biochemistry, Amsterdam UMC location AMC, University of Amsterdam, Amsterdam, The Netherlands
[2] Amsterdam Gastroenterology, Endocrinology, and Metabolism Institute, Amsterdam UMC, The Netherlands
[3] Amsterdam Cardiovascular Sciences Institute, Amsterdam UMC, The Netherlands
[4] Department of Physiology, Amsterdam UMC location VUMC, Free University Amsterdam, Amsterdam, the Netherlands
[5] Tytgat Institute for Liver and Intestinal Research, Amsterdam UMC location AMC, University of Amsterdam, Amsterdam, The Netherlands
[6] Bioinformatics Laboratory, Department of Epidemiology & Data Science, Amsterdam UMC location AMC, Amsterdam, the Netherlands
[7] Department of Experimental Vascular Medicine, Amsterdam UMC location AMC, University of Amsterdam, Amsterdam, The Netherlands
[8] Department of Medicine, Division of Endocrinology & Einthoven Laboratory for Experimental Vascular and Regenerative Medicine, Leiden University Medical Center, Leiden, The Netherlands
[9] Department of Pediatrics, University of Groningen, University Medical Center Groningen, University of Groningen, Groningen, The Netherlands
[10] Department of Laboratory Medicine, University of Groningen, University Medical Center Groningen, Groningen, The Netherlands
[11] European Research Institute for the Biology of Ageing (ERIBA), University of Groningen, University Medical Center Groningen, Groningen, The Netherlands
[12] Gottfried Schatz Research Center, Molecular Biology and Biochemistry, Medical University of Graz, Graz, Austria
[13] BioTechMed-Graz, Graz, Austria
[14] European Center for Angioscience (ECAS), Medical Faculty Mannheim, Heidelberg University, Mannheim, Germany
[15] Institute for Diabetes and Cancer, Helmholtz Center Munich, Neuherberg, Germany
[16] Joint Heidelberg-IDC Translational Diabetes Program, Inner Medicine 1, Heidelberg University Hospital, Heidelberg, Germany
[17] German Center for Diabetes Research (DZD), Neuherberg, Germany

[&] Current address: Department of Biomedical Sciences, University of Copenhagen, Copenhagen, Denmark
[#] These authors contributed equally

[‡] To whom correspondence should be addressed

Sebastian Hendrix, PhD
Department of Medical Biochemistry
Academic Medical Center of the University of Amsterdam
Meibergdreef 9
1105AZ Amsterdam
+31-20-5665161
s.hendrix@amsterdamumc.nl

Noam Zelcer, PhD
Department of Medical Biochemistry
Academic Medical Center of the University of Amsterdam
Meibergdreef 9
1105AZ Amsterdam
+31-20-5665131
n.zelcer@amsterdamumc.nl

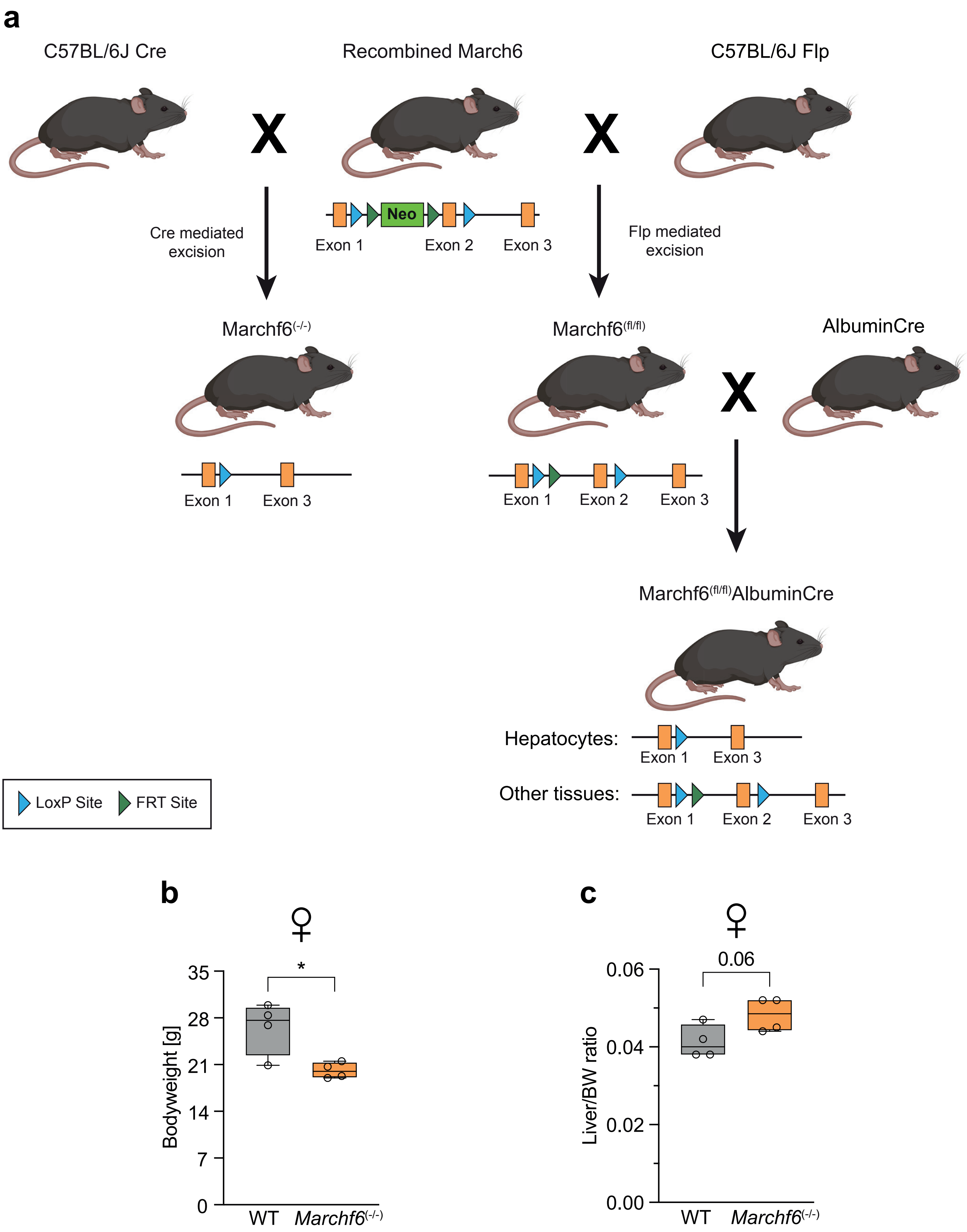
a
C57BL/6J Cre
Recombined March6
C57BL/6J Flp
X
X
Neo
Exon 1
Exon 2
Exon 3
Cre mediated excision
Flp mediated excision
Marchf6(-/-)
Marchf6(fl/fl)
AlbuminCre
Exon 1
Exon 3
Exon 1
Exon 2
Exon 3
Marchf6(fl/fl)AlbuminCre
Hepatocytes:
Exon 1
Exon 3
Other tissues:
Exon 1
Exon 2
Exon 3
LoxP Site
FRT Site
b
♀
*
Bodyweight [g]
35
28
21
14
7
0
WT
Marchf6(-/-)
c
♀
0.06
Liver/BW ratio
0.06
0.04
0.02
0.00
WT
Marchf6(-/-)

Supplementary Figure 1. Mouse model generation and characterization of female *Marchf6*$^{(-/-)}$ mice.(**a**) Schematic representation of the generation of constitutive and hepatocyte-specific *Marchf6* knockout mouse models. (**b**) Bodyweight and (**c**) liver/bodyweight ratio of female WT and *Marchf6*$^{(-/-)}$ mice (n=4/group). Box plots show the median (middle line) 25th, 75th percentile (box), minimum and maximum values (whiskers). * $p<0.05$ as analyzed by two-tailed Welch's t-test.

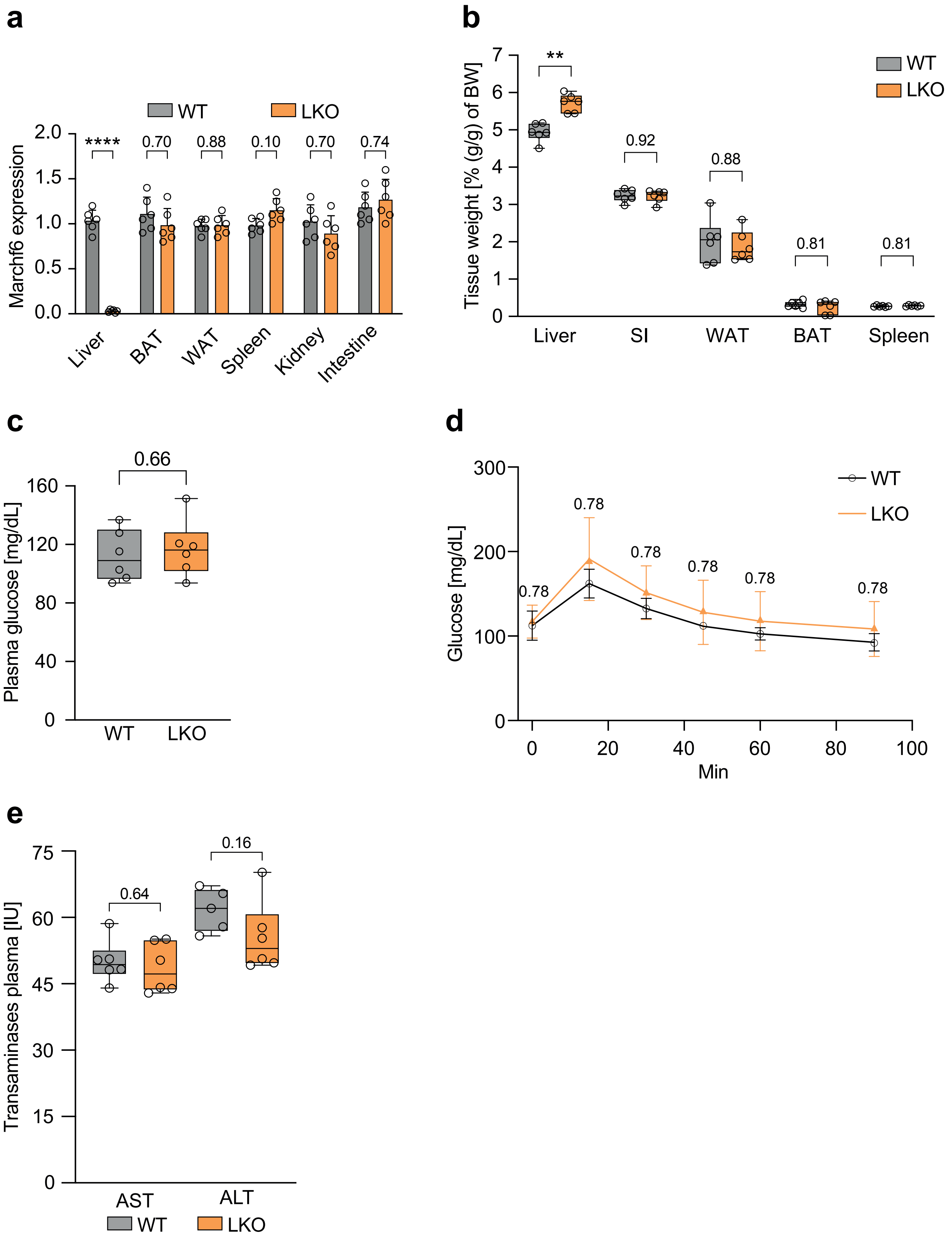

a
WT
LKO
Marchf6 expression
2.0
1.5
1.0
0.5
0.0
****
0.70
0.88
0.10
0.70
0.74
Liver
BAT
WAT
Spleen
Kidney
Intestine
b
Tissue weight [% (g/g) of BW]
7
6
5
4
3
2
1
0
**
0.92
0.88
0.81
0.81
WT
LKO
Liver
SI
WAT
BAT
Spleen
c
Plasma glucose [mg/dL]
0.66
160
120
80
40
0
WT
LKO
d
Glucose [mg/dL]
300
200
100
0
WT
LKO
0.78
0.78
0.78
0.78
0.78
0.78
0
20
40
60
80
100
Min
e
Transaminases plasma [IU]
75
60
45
30
15
0
0.64
0.16
AST
ALT
WT
LKO

Supplementary Figure 2. Characterization of liver-specific *Marchf6* knockout mouse model. (**a**) Male *Marchf6*^{LKO} (n=6) and control littermates (n=6) on standard chow diet were sacrificed at 12 weeks of age and gene expression of *Marchf6* was determined in the indicated tissues. (**b**) Tissue weight of same mice as in (**a**) was determined relative to the total bodyweight of the mice. (**c**) Plasma glucose levels in same mice as in (**a**) were determined after 4 hours of fasting. (**d**) 19-weeks-old female WT and *Marchf6*^{LKO} mice (n=6/group) were fasted for 4 hours and then given a glucose bolus (2g/kg). Blood glucose levels were sampled at the indicated timepoints via the tail vein. (**e**) Plasma transaminases were measured in the same mice as in (**a**) as an indicator for liver damage. (**a**) Bars and (**d**) open circles represent the mean value and the error bars represent the SD. **** p <0.0001 as analyzed by unpaired, two-tailed Welch's t-test with Holm-Šídák correction for multiple comparison. (**b**,**c**,**e**) Box plots show the median (middle line) 25th, 75th percentile (box), minimum and maximum values (whiskers). ** p<0.01, as analyzed by unpaired, two-tailed Welch's t-test with (**b**) Holm-Šídák correction for multiple comparison.

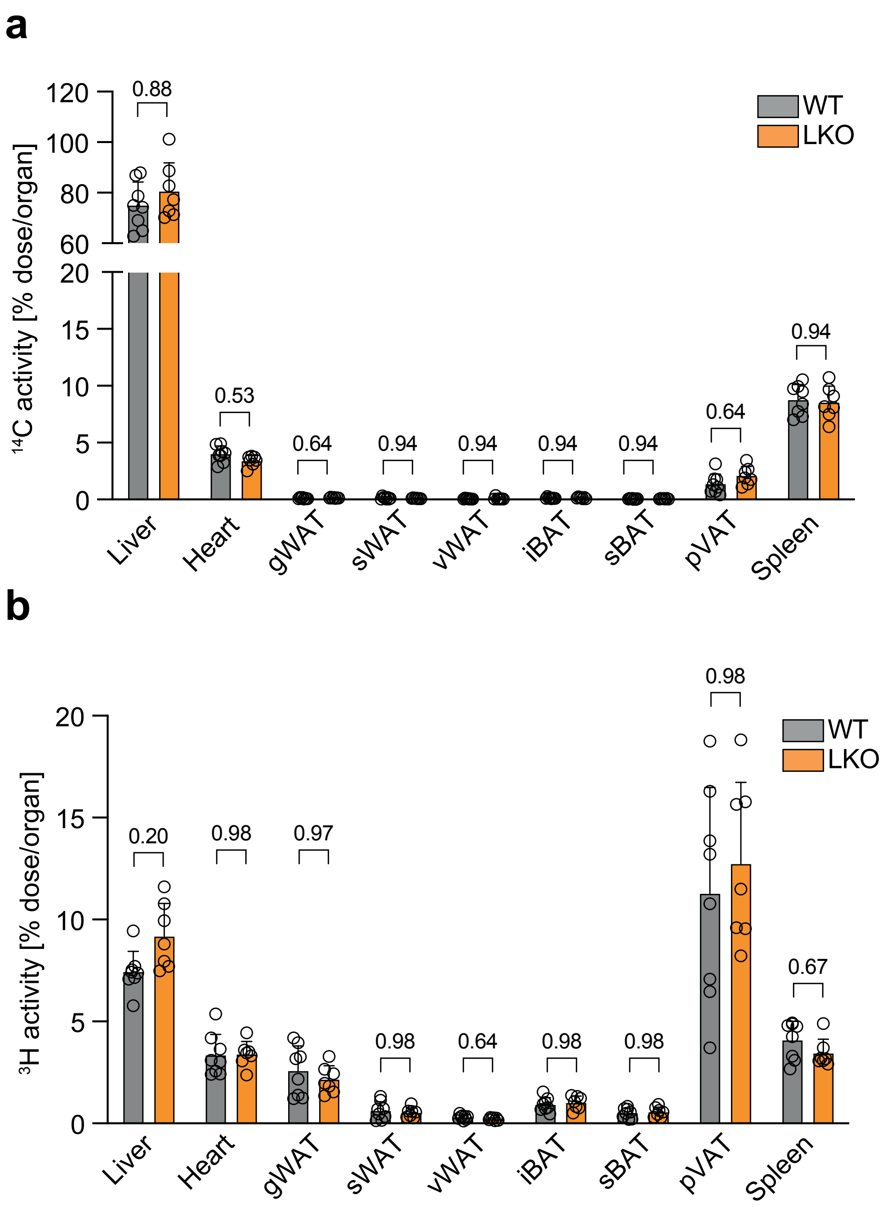
a
120
100
80
60
20
15
10
5
0
^{14}C activity [% dose/organ]
0.88
0.53
0.64
0.94
0.94
0.94
0.94
0.64
0.94
WT
LKO
Liver
Heart
gWAT
sWAT
vWAT
iBAT
sBAT
pVAT
Spleen
b
20
15
10
5
0
^{3}H activity [% dose/organ]
0.20
0.98
0.97
0.98
0.64
0.98
0.98
0.98
0.67
WT
LKO
Liver
Heart
gWAT
sWAT
vWAT
iBAT
sBAT
pVAT
Spleen

Supplementary Figure 3. Uptake of lipoprotein-like particles in different tissues. (**a**,**b**) 15-week-old male WT and *Marchf6*^{LKO} (n=8/group) were injected with [^{3}H]triolein and [^{14}C]cholesteryl oleate labeled TG-rich lipoprotein-like particles. After 20 minutes the mice were euthanized, perfused with ice-cold PBS and organs were harvested. The indicated organs were dissolved and (**b**) ^{3}H and (**c**) ^{14}C activity measured by liquid scintillation. Bars represent the mean value and the error bars represent the SD. Statistical significance was determined by unpaired, two-tailed Welch's t-test with Holm-Šídák correction for multiple comparison.

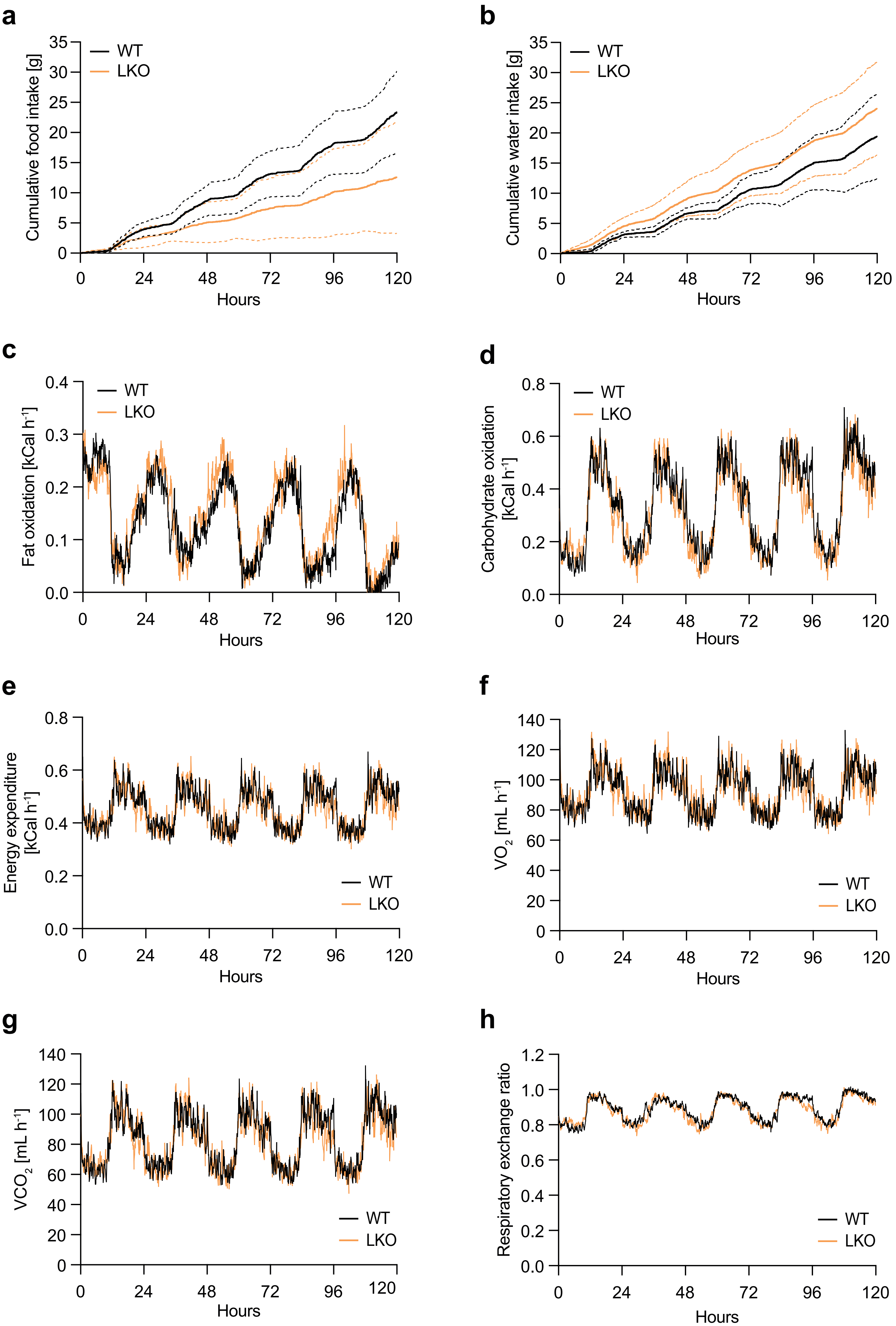
a
Cumulative food intake [g]
WT
LKO
Hours
b
Cumulative water intake [g]
WT
LKO
Hours
c
Fat oxidation [kCal h-1]
WT
LKO
Hours
d
Carbohydrate oxidation [kCal h-1]
WT
LKO
Hours
e
Energy expenditure [kCal h-1]
WT
LKO
Hours
f
VO2 [mL h-1]
WT
LKO
Hours
g
VCO2 [mL h-1]
WT
LKO
Hours
h
Respiratory exchange ratio
WT
LKO
Hours

Supplementary Figure 4. Loss of hepatic *Marchf6* does not affect indirect calorimetric parameters of mice. 11-week-old male WT and *Marchf6*^{LKO} (n=8/group) were individually housed in metabolic cages for 1 week and (**a**) cumulative food intake, (**b**) cumulative water intake, (**c**) fat and (**d**) carbohydrate oxidation, (**e**) energy expenditure, (**f**) O_2 and (**g**) CO2 consumption as well as (**h**) respiratory exchange ratio were continuously measured.

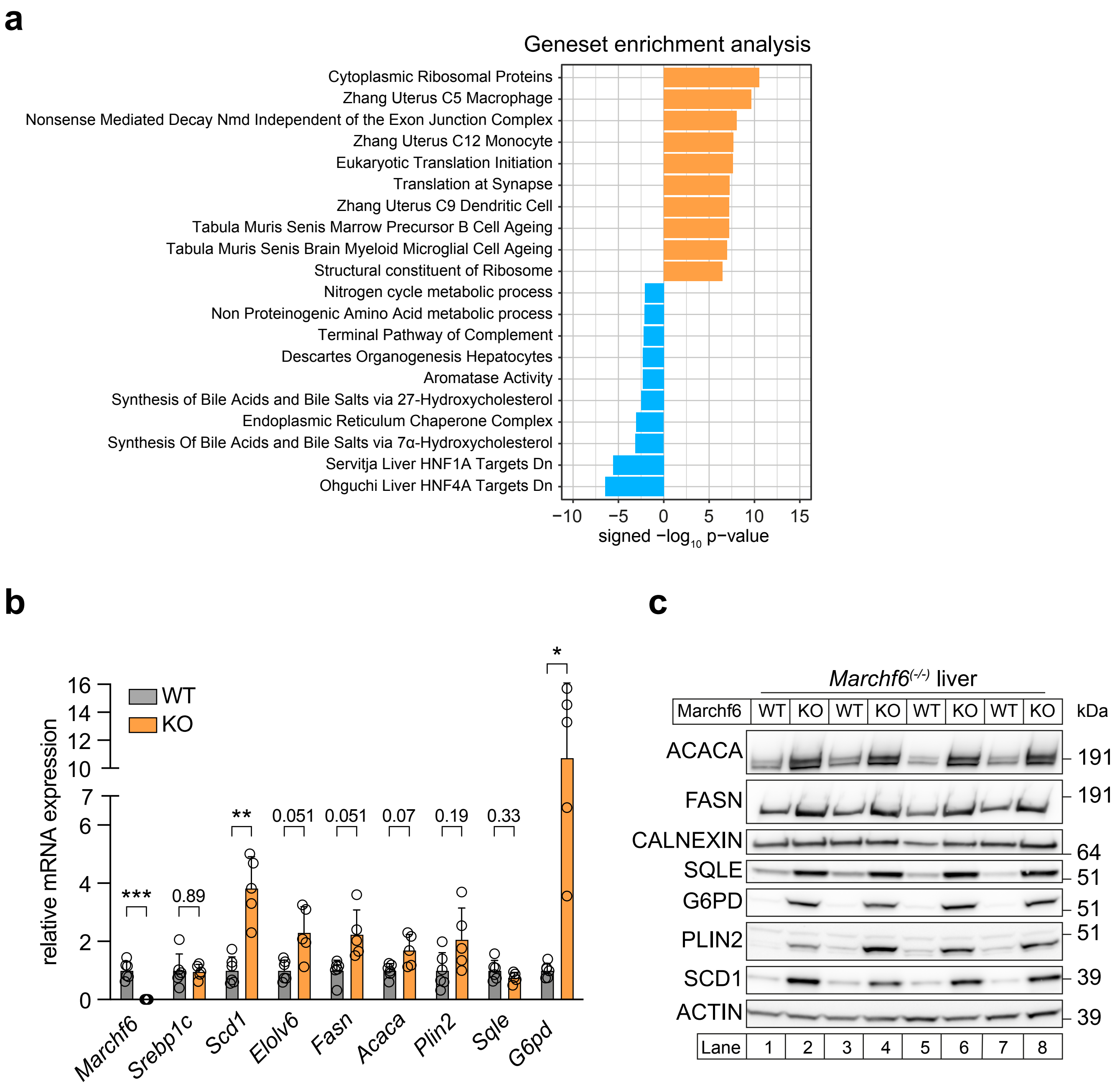
a
Geneset enrichment analysis
Cytoplasmic Ribosomal Proteins
Zhang Uterus C5 Macrophage
Nonsense Mediated Decay Nmd Independent of the Exon Junction Complex
Zhang Uterus C12 Monocyte
Eukaryotic Translation Initiation
Translation at Synapse
Zhang Uterus C9 Dendritic Cell
Tabula Muris Senis Marrow Precursor B Cell Ageing
Tabula Muris Senis Brain Myeloid Microglial Cell Ageing
Structural constituent of Ribosome
Nitrogen cycle metabolic process
Non Proteinogenic Amino Acid metabolic process
Terminal Pathway of Complement
Descartes Organogenesis Hepatocytes
Aromatase Activity
Synthesis of Bile Acids and Bile Salts via 27-Hydroxycholesterol
Endoplasmic Reticulum Chaperone Complex
Synthesis Of Bile Acids and Bile Salts via 7α-Hydroxycholesterol
Servitja Liver HNF1A Targets Dn
Ohguchi Liver HNF4A Targets Dn
−10 −5 0 5 10 15
signed −log10 p−value
b
relative mRNA expression
WT
KO
*** 0.89 ** 0.051 0.051 0.07 0.19 0.33 *
Marchf6 Srebp1c Scd1 Elolv6 Fasn Acaca Plin2 Sqle G6pd
c
Marchf6(-/-) liver
Marchf6 WT KO WT KO WT KO WT KO
kDa
ACACA 191
FASN 191
CALNEXIN 64
SQLE 51
G6PD 51
PLIN2 51
SCD1 39
ACTIN 39
Lane 1 2 3 4 5 6 7 8

Supplementary Figure 5. Expression of lipogenic genes is increased in *Marchf6*$^{(-/-)}$ mice. (**a**) Gene set enrichment analysis of RNAseq experiment shown in main Figure 5A. (**b**) Expression of the indicated genes was assessed by RT qPCR in livers of male WT (n=6) and *Marchf6*$^{(-/-)}$ (n=5) mice. Bars represent the mean value and the error bars represent SD. * p<0.05, ** p<0.01 and *** p <0.001 as analyzed by unpaired, two-tailed Welch's t-test with Holm-Šídák correction for multiple comparison. (**c**) Liver lysates from the same mice as in (**b**) were immunoblotted for the proteins indicated.

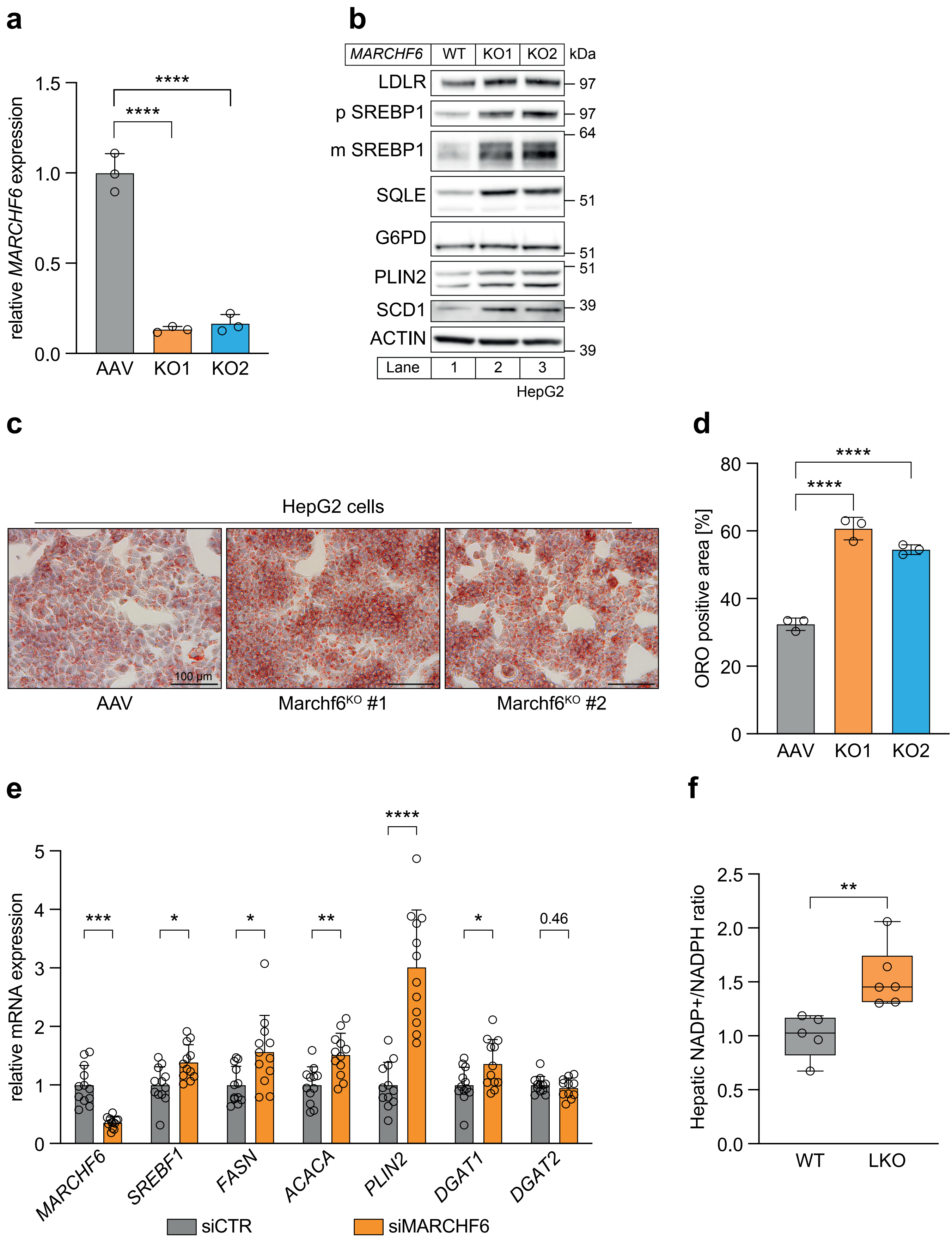

a
relative MARCHF6 expression
1.5
1.0
0.5
0.0
****
****
AAV
KO1
KO2
b
MARCHF6
WT
KO1
KO2
kDa
LDLR
p SREBP1
m SREBP1
SQLE
G6PD
PLIN2
SCD1
ACTIN
97
97
64
51
51
51
39
39
Lane
1
2
3
HepG2
c
HepG2 cells
100 µm
AAV
Marchf6KO #1
Marchf6KO #2
d
ORO positive area [%]
80
60
40
20
0
****
****
AAV
KO1
KO2
e
relative mRNA expression
5
4
3
2
1
0
***
*
*
**
****
*
0.46
MARCHF6
SREBF1
FASN
ACACA
PLIN2
DGAT1
DGAT2
siCTR
siMARCHF6
f
Hepatic NADP+/NADPH ratio
2.5
2.0
1.5
1.0
0.5
0.0
**
WT
LKO

Supplementary Figure 6. Loss of MARCHF6 causes neutral lipid accumulation in HepG2 cells. (**a**) Gene expression of *Marchf6* was determined in AAVS (control) and two independent *Marchf6*^{KO} HepG2 clones. (**b**) Cell lysates of indicated HepG2 clones were lysed and immunoblotted as shown. (**c**) Representative images of ORO-stained HepG2 clones and (**d**) the quantification of ORO positive area is shown. (**b,c**) Representative images from at least 3 independent biological experiments is shown. (**e**) qPCR analysis of the indicated genes in primary human hepatocytes. (**f**) NADP^{+} and NADPH were measured in hepatic tissue of WT and *Marchf6*^{LKO} mice (n=6/group) and the NADP+/NADPH ratio was calculated. (**a**,**d**) Bars represent the mean value and the error bars represent the SD. **** p<0.0001 as analyzed by ordinary one-way ANOVA with Holm-Šídák post hoc analysis to correct for multiple comparisons. (**e**) Bars represent the mean value and the error bars represent SD. * p<0.05, ** p<0.01, *** p <0.001 and **** p<0.0001 as analyzed by unpaired, two-tailed Welch's t-test with Holm-Šídák correction for multiple comparison. (**f**) Box plots show the median (middle line) 25th, 75th percentile (box), minimum and maximum values (whiskers). ** p<0.01 as analyzed by unpaired, two-tailed Welch's t-test.
