# Supplementary material for: Loss of the E3 ubiquitin ligase MARCHF6 alters hepatic lipid metabolism and drives spontaneous hepatosteatosis
Source: Mol Metab. 2026 May 2;108:102379. doi: 10.1016/j.molmet.2026.102379 (PMC13194171; doi:10.1016/j.molmet.2026.102379)
Supplement: Multimedia component 2 [file mmc2.docx]

| **Chemicals** | **Source** | **Identifier** |
| --- | --- | --- |
| 6-aminonicotinamide | Sigma-Aldrich | A68203 |
| Acetic Acid [1,2-14C] Na salt | American Radiolabled Chemicals | ACR 0173 |
| BCA assay | Thermo Fisher | 23225 |
| BODYPI | Thermo Fisher | D3922 |
| BSA | Sigma | #10735086001 |
| CaCl | Sigma | C4909-500G |
| cDNA synthesis kit | Biotool | #B24403 |
| Chloroform | VWR | 83627.209 |
| Cholesteryl oleate [Oleate-1-14C] | Revity | NEC638050UC |
| citric acid monohydrate | Carl Roth, | 3958 |
| Collagen | Sigma-Aldrich | C8897 |
| Collagenase type IV | Merck | C5138-1G |
| D-glucose | Sigma | G7528-250G |
| DAPI | Thermo Fisher | D1306 |
| Dexamethasone | Sigma | D4902-100MG |
| Dexamethasone | Sigma | 102400400 |
| Direct-zol RNA MiniPrep kit | Zymo Research | #R2052 |
| DMEM | Thermo Fisher | #31966 |
| FA free BSA | Biowest | P6156 |
| FBS | Thermo Fisher | A5256701 |
| Glycerol trioleate [9,10(N)-3H] | Revity | NET431L005MC |
| HBSS | Gibco | 14175-053 |
| HEPES | Sigma | H3375 500G |
| Horse Serum | Thermo Fisher | 16050130 |
| iBlot 2 Transfer Stacks | Thermo Fisher | IB23001 |
| Insulin-Transferrin-Selenium | Gibco | 41400045 |
| iScript reverse transcription | BioRad | 170-8891 |
| Isopentane | Merck | 106056.1 |
| Ketamine | Alfasan | 30040.5 |
| Methanol | Biosolve | 0013680502BS |
| Mevalonate | Sigma | #M4667 |
| Milk | Elk |  |
| MOPS Buffer | Thermo Fisher | NP0001-02 |
| MOWIOL 488 | Merck | 81381 |
| NaCl | Boom | pROD2402606 |
| NaHCO3 | Merck | 1.06529.0500 |
| NB598 | MedChemExpress | HY-16343C |
| NuPAGE 4-12% Bis-Tris Protein Gels | ThermoFisher | NP0321BOX |
| NuPAGE LDS Sample Buffer (4X) | ThermoFisher | NP0007 |
| OilRed O | Sigma | O0625-25G |
| Paraformaldehyde | Merck | 1.04005.100 |
| Paraformaldehyde | Merck | 1.04005.100 |
| PBS | Fresenius Gabi | M090001/02NL |
| Penicillin-Streptomycin | Invitrogen | 15140-122 |
| Phenylmethylsulfonyl fluoride | Sigma | 78830-5G |
| Poloxamer 407 | Merck | 16758-250G |
| Protease inhibitors | Roche | #P8340 |
| Protease inhibitors | Cell Signaling | 5872S |
| Puromycin | Sigma | P7255-100MG |
| Qiagen DNA isolation kit | Qiagen | #51304 |
| RIPA buffer | Boston Biochem | #BP-115 |
| RNAiMAX | Thermo Fisher | 13.778-150 |
| SensiFAST SYBR | Bioline | #BIO-98020 |
| Simvastatin | Calbiochem | #567021 |
| Sodium acetate-1-13C | Sigma | 279293-1G |
| Sodium bicarbonate | Gibco | 25080094 |
| ß-Mercaptoethanol | Sigma | M3148 |
| Tamoxifen | Sigma | T5648-1G |
| TissueTek | Sakura Finetek | 4583 |
| TriReagent | Sigma | T9424 |
| Tris-HCL | Biomol | 8003.5 |
| trisodium citrate, dehydrated, | Carl Roth, | 3580 |
| Trypan Blue | Thermo Fisher | 15250-061 |
| Trypsin | Merck | T7575-1KT |
| Tween-20 | Sigma | P1379 |
| Williams E medium | Thermo Fisher | 12551032 |
| Xylazine | Dechra | 615319 |
