# Supplementary material for: Loss of the E3 ubiquitin ligase MARCHF6 alters hepatic lipid metabolism and drives spontaneous hepatosteatosis
Source: Mol Metab. 2026 May 2;108:102379. doi: 10.1016/j.molmet.2026.102379 (PMC13194171; doi:10.1016/j.molmet.2026.102379)
Supplement: Multimedia component 4 [file mmc4.docx]

| Antibody | Vendor | Identifier | Dilution | Used for |
| --- | --- | --- | --- | --- |
| ACACA | Cell Signaling | CS36765 | 1:1000 | WB |
| Calnexin | Sigma | C4731 | 1:1000 | WB |
| FASN | Cell Signaling | CS31805 | 1:1000 | WB |
| Flag-Tag | Merck | F1804-50UG | 1:1000 | IP |
| G6PD | Abcam | ab210702 | 1:1000 | WB |
| Goat anti-Mouse IgG | Invitrogen | A28177 | 1:2500 | WB |
| Goat anti-Rabbit IgG | Invitrogen | A27036 | 1:2500 | WB |
| LDLR | Biovision | #3839 | 1:1000 | WB |
| PLIN2 | Abcam | ab78920 | 1:1000 | WB |
| SCD1 | Cell Signaling | 2438S | 1:1000 | WB |
| SQLE | Proteintech | #12544-1-AP | 1:1000 | WB |
| SREBP1 | EDM Millipore | MABS1987; Clone 20B12 | 1:1000 | WB |
| ß-actin | EDM Millipore | #MAB1501 | 1:2500 | WB |
| V5-Tag | Invitrogen | 64-0705 | 1:1000 | IP |
| MARCHF6 antibodies tested | | | | |
| MARCHF6 | Abnova | H00010299-A01 | 1:500, 1:1000 | WB |
| MARCHF6 | Bethyl Lab | A304-171A | 1:500, 1:1000 | WB |
| MARCHF6 | Origene | TA303324 | 1:500, 1:1000 | WB |
| MARCHF6 | Eurogentec | Raised in rabbits | 1:500, 1:1000 | WB |
