# Supplementary material for: Loss of the E3 ubiquitin ligase MARCHF6 alters hepatic lipid metabolism and drives spontaneous hepatosteatosis
Source: Mol Metab. 2026 May 2;108:102379. doi: 10.1016/j.molmet.2026.102379 (PMC13194171; doi:10.1016/j.molmet.2026.102379)
Supplement: Multimedia component 5 [file mmc5.docx]

| **Gene** | **Species** | Primer | |
| --- | --- | --- | --- |
| Marchf6 | mus musculus | fw | TGTCGGTCAGAAGGAACACC |
|  |  | rev | TCCGACTATGTTTCAACCACTGA |
| Srebf1 | mus musculus | fw | CCGGCTATTCCGTGAACATC |
|  |  | rev | ATCCAAGGGCATCTGAGAACTC |
| 36b4 | mus musculus | fw | AGATGCAGCAGATCCGCA |
|  |  | rev | GTTCTTGCCCATCAGCACC |
| Acaca | mus musculus | fw | GGAGATGTACGCTGACCGAG |
|  |  | rev | TACCCGACGCATGGTTTTCA |
| Fasn | mus musculus | fw | GCTGCGGAAACTTCAGGAAAT |
|  |  | rev | AGAGACGTGTCACTCCTGGACTT |
| Scd1 | mus musculus | fw | CCAAGCTGGAGTACGTCTGG |
|  |  | rev | CAGAGCGCTGGTCATGTAGT |
| Cpt1a | mus musculus | fw | GGACTCCGCTCGCTCATT |
|  |  | rev | GAGATCGATGCCATCAGGGG |
| Lpcat3 | mus musculus | fw | GGCCTCTCAATTGCTTATTTCA |
|  |  | rev | AGCACGACACATAGCAAGGA |
| Lipe | mus musculus | fw | AGACACCAGCCAACGGATAC |
|  |  | rev | ATCACCC TCGAAGAAGAGCA |
| Pnpla2 | mus musculus | fw | TGTGGCCTCATTCCTCCTAC |
|  |  | rev | TCGTGGATGTTGGTGGAGCT |
| Dgat1 | mus musculus | fw | TCCGTCCAGGGTGGTAGTG |
|  |  | rev | TGAACAAAGAATCTTGCAGACGA |
| Dgat2 | mus musculus | fw | AAACAGCTGCAGGTCATCTCA |
|  |  | rev | TCCTGCCACCTTTCTTGGGC |
| Mogat2 | mus musculus | fw | TACAGCTTTGGCCTCATGC |
|  |  | rev | AGGGCTGTGGTGTCATCTG |
| Plin2 | mus musculus | fw | GGCGTCTCTTTTCTCCAGGA |
|  |  | rev | CGGATCCACTACTGCTGCTG |
| Elovl6 | mus musculus | fw | GCTGACTCTTGCCGTCTTCA |
|  |  | rev | GTGTCACCTAGTTCGGGTGC |
| Sqle | mus musculus | fw | GCCTCTCAGAATGGTCGTCT |
|  |  | rev | CGCATCTCCCAGAATAAGGA |
| Ldlr | mus musculus | fw | AGGCTGTGGGCTCCATAGG |
|  |  | rev | TGCGGTCCAGGGTCATCT |
| Hmgcs | mus musculus | fw | GGTCTGATCCCCTTTGGTG |
|  |  | rev | TGTGAAGGACAGAGAACTGTGG |
| Srebf2 | mus musculus | fw | ACCTAGACCTCGCCAAAGGT |
|  |  | rev | GCACGGATAAGCAGGTTTGT |
| Hmgcr | mus musculus | fw | TCTGGCAGTCAGTGGGAACTATT |
|  |  | rev | CCTCGTCCTTCGATCCAATTT |
| Idol | mus musculus | fw | AGGAGATCAACTCCACCTTCTG |
|  |  | rev | ATCTGCAGACCGGACAGG |
| Abca1 | mus musculus | fw | CTCTTCATGACTCTAGCCTGGA |
|  |  | rev | ACACAGACAGGAAGACGAACAC |
| MARCHF6 | homo sapiens | fw | GGAGGAAGATGACGCTGGT |
|  |  | rev | GCATTCCAATTCATGTCATCC |
